# Structural Disorder within *Henipavirus* Nucleoprotein and Phosphoprotein: From Predictions to Experimental Assessment

**DOI:** 10.1371/journal.pone.0011684

**Published:** 2010-07-21

**Authors:** Johnny Habchi, Laurent Mamelli, Hervé Darbon, Sonia Longhi

**Affiliations:** Architecture et Fonction des Macromolécules Biologiques, UMR 6098 CNRS et Universités Aix-Marseille I et II, Campus de Luminy, Marseille, France; Griffith University, Australia

## Abstract

*Henipaviruses* are newly emerged viruses within the *Paramyxoviridae* family. Their negative-strand RNA genome is packaged by the nucleoprotein (N) within α-helical nucleocapsid that recruits the polymerase complex made of the L protein and the phosphoprotein (P). To date structural data on *Henipaviruses* are scarce, and their N and P proteins have never been characterized so far. Using both computational and experimental approaches we herein show that *Henipaviruses* N and P proteins possess large intrinsically disordered regions. By combining several disorder prediction methods, we show that the N-terminal domain of P (PNT) and the C-terminal domain of N (N_TAIL_) are both mostly disordered, although they contain short order-prone segments. We then report the cloning, the bacterial expression, purification and characterization of *Henipavirus* PNT and N_TAIL_ domains. By combining gel filtration, dynamic light scattering, circular dichroism and nuclear magnetic resonance, we show that both N_TAIL_ and PNT belong to the premolten globule sub-family within the class of intrinsically disordered proteins. This study is the first reported experimental characterization of *Henipavirus* P and N proteins. The evidence that their respective N-terminal and C-terminal domains are highly disordered under native conditions is expected to be invaluable for future structural studies by helping to delineate N and P protein domains amenable to crystallization. In addition, following previous hints establishing a relationship between structural disorder and protein interactivity, the present results suggest that *Henipavirus* PNT and N_TAIL_ domains could be involved in manifold protein-protein interactions.

## Introduction

Hendra virus (HeV), the first known member of the genus *Henipavirus* within the *Paramyxoviridae* family, emerged in 1994 as the causative agent of a sudden outbreak of acute respiratory disease in horses in Brisbane, Australia. Nipah virus (NiV), the second known member of the genus *Henipavirus*, came to light as the etiologic agent of an outbreak of disease in pigs and humans in Malaysia in 1998 through 1999. The initial NiV outbreak in Malaysia resulted in 265 human cases of encephalitis, including 105 deaths. The virus reemerged in Bangladesh in 2001 and outbreaks of encephalitis caused by NiV have occurred in that country almost every year since, with a case fatality rate approaching 75% (see [1] and references therein cited). Later on, fruit-eating bats were shown to be the natural reservoir of both viruses (see [2] and references therein cited).

Although the genome of HeV and NiV shares the same overall organization of members of the *Paramyxovirinae* subfamily, a few distinctive properties, including their much larger size, led to the creation of the *Henipavirus* genus to accommodate these newly emerged zoonotic viruses [3]. Currently this genus contains two virus species and a number of strains isolated from humans, bats, horses and pigs over a wide geographic area and during a period of 10 years. Noteworthy, recently Henipaviruses have also been found outside Australia and Asia, thus extending the region of potential endemicity of one of the most pathogenic virus genera known in humans [2]. The susceptibility of humans, the wide host range and interspecies transmission and the absence of therapeutics agents led to the classification of HeV and NiV as biosecurity level 4 (BSL4) pathogens [4].

*Henipaviruses* particles are pleomorphic and enveloped. Their negative-stranded, non-segmented RNA genome is encapsidated by the nucleoprotein (N) within α-helical nucleocapsid that has the characteristic herringbone-like structure typically observed in other *Paramyxoviridae* members including measles virus (MeV) [5], [6], [7], [8] and Sendai virus (SeV) [9], [10]. This helical nucleocapsid, rather than naked RNA, is the substrate used by the polymerase complex during both transcription and replication. Minigenome replicon studies showed that *Henipavirus* N, P and L proteins are necessary and sufficient to sustain replication of viral RNA [11]. By analogy with other *Paramyxoviridae* members, the polymerase complex is assumed to consist of the L protein and of the phosphoprotein (P), with this latter serving as a tethering anchor for the recruitment of L onto the nucleocapsid template.

The genome organization of *Henipaviruses* resembles that in the *Respirovirus* and *Morbillivirus* genera. The extra length of the *Henipavirus* genome mainly arises from additional unique, long untranslated sequences at the 3′ end of five of the six genes. Despite their much larger genome size, the genome length is divisible by six and reverse genetics studies confirmed that NiV does obey the “rule of six”, i.e. the genome length must be a multiple of six to replicate efficiently [11]. Overall, the proteins of *Henipaviruses* are typical of those of the *Paramyxovirinae* subfamily, with the exception of the P protein that is significantly larger than cognate proteins in the subfamily. Despite this difference in size, the organization of *Henipavirus* P proteins closely resembles that of other members in the subfamily. Indeed, beyond the P protein, that is translated by an mRNA co-linear with genomic RNA, the P gene of *Paramyxovirinae* also encodes the V and W proteins that are produced upon addition of either one or two non-templated G residues at the editing site of the P messenger (see [1] and references therein cited). The P, V and W proteins are therefore identical for the first 404 (HeV) or 406 (NiV) residues. A C protein is also encoded by the 5′ end of the gene in an overlapping reading frame and is produced by an internal translational initiation mechanism, which is common to other members of *Paramyxovirinae*, except for *Rubulaviruses*. The P, V and C proteins predicted from the coding region of the P gene are indeed present in HeV-infected cells. As for NiV, although no formal proof indicates that the W protein is expressed, this latter displays anti-interferon signaling activity when expressed from cloned genes [12]. In addition, in this latter virus, the C, V and W proteins were shown to inhibit minigenome replication [13].

So far, structural and molecular information on *Henipavirus* proteins is scarce and limited to their surface proteins, where crystallographic studies led to the determination of the 3D structure of *Henipavirus* fusion (F) and attachment (G) proteins [14], [15], [16], [17].

Previous computational and experimental studies carried out by our laboratory pointed out that MeV N and P possess large (up to 230 residues) intrinsically disordered regions (IDRs) [6], [18], [19], [20], [21], [22], [23], [24], [25]. Using computational approaches, we then extended these findings to the N and P proteins of *Paramyxovirinae* members [26] and showed that the presence of IDRs is a conserved feature within the replicative complex of these viruses.

IDRs are regions that lack highly populated constant secondary and tertiary structure under physiological conditions and in the absence of a partner/ligand [27] (for recent reviews on intrinsically disordered proteins – IDPs - see [28], [29]). Intrinsically disordered proteins show an extremely wide diversity in their structural properties: indeed they can attain extended conformations (random coil-like) or remain globally collapsed (molten globule-like), where the latter possess regions of fluctuating secondary structure. Conformational and spectroscopic analyses showed that random coil-like proteins (RCs) can be subdivided in their turn into two major groups. While the first group consists of proteins with extended maximum dimensions typical of random coils with no (or little) secondary structure, the second group comprises the so-called premolten globules (PMGs), which are more compact (but still less compact than globular or molten globule proteins) and conserve some residual secondary structure [30], [31], [32], [33], [34], [35].

As a first step towards the structural characterization of *Henipavirus* N and P proteins, we herein describe the results of a thorough computational analysis that shows that *Henipaviruses N and P* proteins possess a modular organization consisting of large (up to 400 residues) unstructured regions alternating with structured regions. We show that the N-terminal domain of P (PNT) and the C-terminal domain of N (N_TAIL_) possess the peculiar sequence features that typify IDRs. We also report the cloning, bacterial expression, purification and characterization of *Henipavirus* N_TAIL_ and PNT domains. Using different, complementary biochemical and biophysical methods, we confirmed the predicted disordered nature of *Henipavirus* N_TAIL_ and PNT and show that they belong to the PMG subfamily within the class of IDPs.

## Results

### Disorder predictions and modular organization of *Henipavirus* N and P

We first analyzed the amino acid sequences of *Henipavirus* N and P proteins using the MeDor metaserver for the prediction of disorder [36]. In the graphical output generated by MeDor, disordered regions, as predicted by the various predictors, are shown along with the HCA plot of the query sequence.

**Figure 1:**
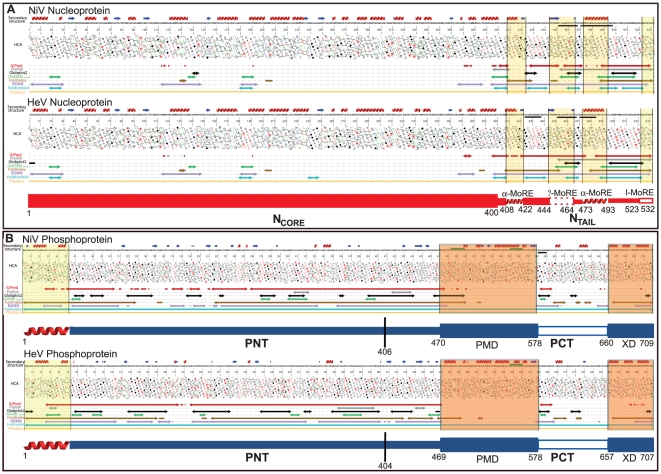
Disorder prediction and modular organization of *Henipavirus* N and P. MeDor ouputs (available also as supplementary material) and proposed modular organization of *Henipavirus* N (**A**) and P (**B**) proteins. The sequences are represented as single, continuous horizontal lines below the predicted secondary structure elements. Low sequence complexity regions (NiV N: aa 451–467 and 470–497; HeV N: aa 423–437, 451–467 and 470–483; NiV P: aa 580–589) are shown by a black bar below the sequence. Coiled-coil regions (NiV P: aa 482–497 and 548–562; HeV P: aa 546–560), as obtained using a 14-residues window, are indicated by a green bar above the sequence. Below the sequences are shown the HCA plots and the predicted regions of disorder that are represented by bi-directional arrows. Regions highlighted in yellow and in orange correspond to either putative Molecular Recognition Elements (MoREs) or structured regions (PMD and XD) within PCT, respectively. Structured and disordered regions are represented by large and narrow boxes, respectively. The linker region connecting PMD and XD is shown by an empty, narrow box to indicate its mixed nature. The vertical line separating PNT and PCT is located at the border between the region shared by P and V and the region unique to P (see text). The hydrophobic, α-helical region at the N-terminus of P is highlighted.

As shown in **Fig. 1A**, both nucleoproteins consist of a large (400 residues) N-terminal region (referred to as N_CORE_), which is consistently predicted to be ordered by the various predictors and that is enriched in hydrophobic clusters, and of a C-terminal region (referred to as N_T**AIL**_) that is predicted to be disordered by most predictors and that is depleted in hydrophobic clusters. Interestingly, two or three low sequence complexity regions, which are hallmarks of structural disorder [37], were found within the NiV and HeV N_T**AIL**_, respectively (**Fig. 1**). Besides, analysis of a multiple sequence alignment among *Henipavirus*, *Morbillivirus* and *Respirovirus* N proteins (see supplementary **Fig. S1**) revealed a significant sequence divergence in the N_TAIL_ region, in agreement with earlier observations pointing out a higher sequence variability in disordered regions as compared to structured ones [38]. Although the N_T**AIL**_ region is mostly disordered, four short regions possessing each a hydrophobic cluster and/or a predicted (α or β) secondary structure element, are found (see **Fig. 1A**).

The P protein of both HeV and NiV displays a more complex organization, with regions of predicted disorder alternating with structured regions (**Fig. 1B**). With the only exception of the first 50 residues, which are predicted to be ordered, the P protein of both viruses possesses a spectacularly large N-terminal region of about 400 residues that is depleted in hydrophobic clusters, that is predicted to be disordered by most predictors and that possesses very few predicted secondary structure elements, a feature typifying protein regions with “no ordered regular structure” [39]. This region of predicted disorder encompasses the region shared by the P, V and W proteins (referred to as P N-Terminal, PNT) and further extends by approximately 65 residues towards the C-terminus (see **Fig. 1B**). A region predicted to be structured, and whose HCA plot is reminiscent of coiled-coil regions follows downstream (see **Fig. 1B**) (for examples of such patterns see Fig. 2 in [40], Fig. 2 in [41] and Fig. 4 in [26]). Notably, one or two coiled-coils are predicted within this region of the HeV (aa 546–560) or NiV (aa 482–497 and 548–562) P protein, respectively. In further support of the occurrence of a coiled-coil within this region, the majority of the best PDB hits, as provided by the 3D-PSSM server, are coiled–coils (for an example see pdb code 1sfc). By analogy with cognate *Paramyxovirinae* P proteins [26], this region could correspond to a putative P multimerization domain (PMD), although so far no direct information is available on the oligomeric state of the *Henipavirus* P protein.

The C-terminal region of both P proteins is predicted to be structured and to adopt an α-helical conformation, as judged based on the occurrence of three predicted α-helices (see **Fig. 1B**). Again, by analogy with the P protein of other *Paramyxovirinae* members, this globular region has been assumed to be the counterpart of the X domain (XD), the structure of which consists of a triple α-helical bundle [42], [43], [44].

PMD and XD are connected by a mixed linker region consisting of *(i)* a short disordered segment (aa 579–590), which also corresponds to a low complexity region in NiV P (see **Fig. 1B**), *(ii)* a region with a borderline order (aa 590–640) and finally *(iii)* a short disordered segment.

### Sequence properties of *Henipavirus* N_TAIL_ and PNT

**Figure 2:**
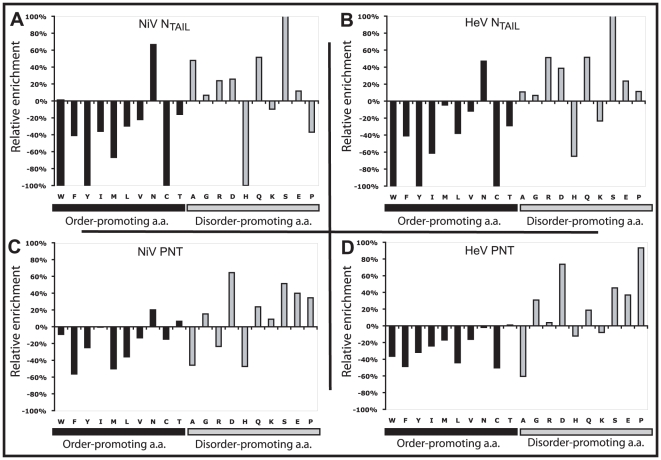
Amino acid composition analysis of *Henipavirus* N and P proteins. Deviation in amino acid composition from the Swiss-PROT data base of NiV N_TAIL_ (**A**), HeV N_TAIL_ (**B**), NiV PNT (**C**) and HeV PNT (**D**). The relative enrichment in disorder promoting (black bars) and order-promoting (grey bars) residues is shown. Residues have been ordered on the x axis according to the TOP-IDP flexibility index as described in [138].

We compared the sequence composition of *Henipavirus* N_TAIL_ and PNT to that of proteins within the SWISS-PROT database (**Fig. 2**). Both *Henipavirus* N_TAIL_ proteins (**Fig. 2A, B**) have a peculiar amino acid composition, in that they are depleted in most “order promoting” residues (W, C, F, Y, I, V, L) and enriched in most “disorder promoting” residues (A, R, Q, S, P and E), as already described for the cognate N and P regions of other *Paramyxovirinae* members [6], [19], [26] and, more generally, for IDPs [30]. A similar, though less pronounced, compositional bias is observed for *Henipavirus* PNT (**Fig. 2 C, D**). Conversely, *Henipavirus* N_CORE_, PMD and XD regions do not display any significant overall relative enrichment or depletion with respect with the average amino acid composition in the SWISS-PROT database (data not shown).

**Figure 3:**
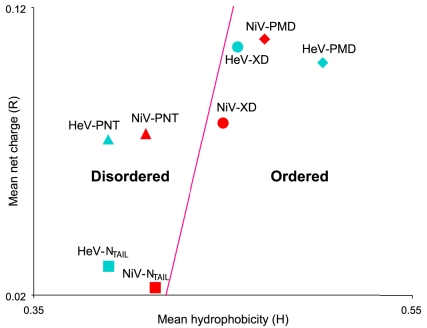
Charge-hydropathy plot of *Henipavirus* N and P domains. The mean net charge (R) is plotted against the mean hydrophobicity (H). In the left part of the CH plot, a protein is predicted to be intrinsically disordered, whereas it is predicted to be structured if it falls in the right part of it (see Materials and Methods).

Moreover, *Henipavirus* N_T**AIL**_ and PNT are predicted to be disordered by the method based on the mean hydrophobicity/mean net charge ratio [45], whereas PMD and XD are predicted to be ordered (**Fig. 3**).

In order to experimentally confirm the disorder predictions, we have expressed, purified and characterized the large regions of predicted disorder of *Henipavirus* N and P proteins. Taking into account the fact that the PNT region is shared by the P, V and W proteins, we reasoned that it is likely to constitute an independent, functional domain. We therefore decided to clone and characterize this latter region (residues 1–404 for HeV and 1–406 for NiV), rather than the entire disordered N-terminal P region extending up to residue 470 (see **Fig. 1B**). As for the N protein, we focused our efforts on the N_T**AIL**_ region.

### Expression and purification of *Henipavirus* N_TAIL_ and PNT domains

We cloned the DNA fragments of the *Henipavirus* N and P genes encoding N_TAIL_ and PNT into the pDest14 expression plasmid that allows expression in *E. coli* of recombinant proteins under the control of the T7 promoter. Primers were designed so as to lead to constructs encoding the N_TAIL_ and PNT domains with either an N-terminal or a C-terminal histidine tag, respectively. The *E. coli* Rosetta [DE3] pLysS strain (Novagen) was used for the expression of the constructs.

**Figure 4:**
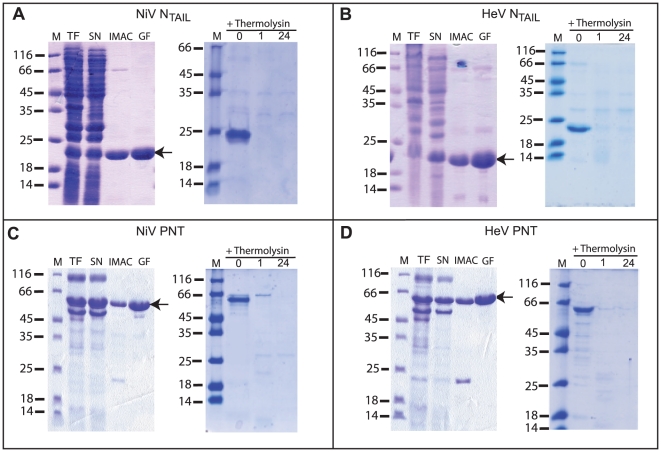
Purification of *Henipavirus* N_TAIL_ and PNT proteins from *E. coli* and digestion with thermolysin. Coomassie blue staining of SDS-PAGE showing the bacterial lysates (total fractions (TF), the clarified supernatants (soluble fractions) (SN), the eluents from Immobilized Metal Affinity Chromatography (IMAC) and the eluents from Gel Filtration (GF) for the four recombinant proteins. The arrows show the final purified products. The extent of thermolysin digestion of the purified proteins at different time intervals (0, 1h and 24h) is also shown. M: molecular mass markers.

Both PNT and N_T**AIL**_ proteins were recovered from the soluble fraction of bacterial lysates (**Fig. 4**, lanes SN) and were purified to homogeneity (>95%) in two steps: Immobilized Metal Affinity Chromatography (IMAC) and gel filtration (**Fig. 4**). The identity of all the recombinant products was confirmed by mass spectrometry analysis of the tryptic fragments obtained after digestion of the purified proteins excised from SDS-polyacrylamide gels (data not shown). Both N_T**AIL**_ proteins display an abnormally slow migration in SDS-PAGE with an apparent molecular mass of 20 kDa (expected MM 15 kDa) (**Fig. 4 A, B**). A similar aberrant electrophoretic mobility is observed for PNT proteins (**Fig. 4 C, D**), where these latter migrate with an apparent molecular mass of approximately 60 kDa (expected molecular mass of 45 KDa). Noteworthy, mass spectrometry analysis confirmed that the recombinant products possess the expected molecular mass (see supplementary **Figs. S2, S3, S4, S5**). This abnormal behavior is therefore likely to be ascribed to a rather high content of acidic residues, as already been observed in the case of the intrinsically disordered MeV PNT [18] and N_T**AIL**_ domains [6], [19], and, more generally, in other IDPs [46]. Indeed, because of their biased amino acid composition, often leading to enrichment in negatively charged residues, IDPs bind less SDS than usual. As a result their apparent molecular mass is often 1.2–1.8 times higher than the real one calculated from sequence data or measured by mass spectrometry [46].

In the case of NiV PNT, a minor lower band is also observed in the final product (see **Fig. 4C**, lane GF). Mass spectrometry analysis of the tryptic fragments obtained after digestion of this minor band showed that it corresponds to a degradation product of PNT.

### Protease sensitivity of *Henipavirus* N_TAIL_ and PNT

*Henipavirus* PNT and N_TAIL_ were found to be highly sensitive to proteolysis, a property that constitutes a hallmark of structural disorder (see [47] and references therein cited). Indeed, globular proteins are preferentially cleaved at exposed and flexible loops only and almost never within secondary structure elements [48], [49]. The use of a protease with broad substrate specificity, such as thermolysin allows the identification of cleavage sites solely on the basis of the flexibility of the protein substrate. In order to assess the extent of protease sensitivity, we submitted *Henipavirus* PNT and N_TAIL_ to digestion by thermolysin. As shown in **Fig. 4**, all the proteins are readily degraded by thermolysin after one hour incubation, a behavior that is consistent with the lack of a packed core and with an overall solvent accessibility of *Henipavirus* PNT and N_TAIL_. Conversely, lysozyme was shown to be resistant to proteolysis even after an incubation period as long as 24 hours (see supplementary **Fig. S6**).

### Size-exclusion chromatography analyses of *Henipavirus* PNT and N_TAIL_

**Figure 5:**
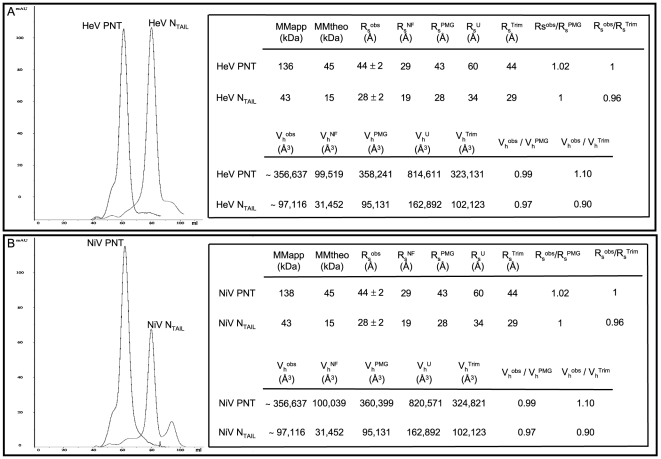
Hydrodynamic properties of *Henipavirus* N_TAIL_ and PNT proteins. Elution profile of HeV (**A**) and NiV (**B**) N_TAIL_ and PNT proteins from gel filtration showing the main peak. The insets show the molecular masses deduced from the calibration column (MMapp), the deduced values of the Stokes radii (R_S_^obs^) and of the hydrodynamic volume (V_h_^obs^), as well as the expected molecular masses (MMtheo) and the expected values for the various conformational states (see text). ^NF^: natively folded; ^U^: fully unfolded; ^PMG^: premolten globule; ^Trim^: trimeric form.

Since the elution volume of a protein from a gel filtration column depends on its hydrodynamic properties, we used size-exclusion chromatography (SEC) to infer the hydrodynamic properties of *Henipavirus* PNT and N**_T_**_AIL_ proteins (**Fig. 5**). *Henipavirus* PNT and N**_TAIL_** are eluted form the gel filtration column as sharp peaks with an apparent molecular mass (MMapp) well above the expected one (MMtheo). These large values of apparent molecular mass indicate that these proteins are not compatible with a monomeric, globular structure (**Fig. 5**). Rather, such large values of apparent molecular mass can be attributed either to trimerization or to an extended conformation with low compactness of the polypeptide chain typical of IDPs [50]. Note that these very high values of molecular mass can't be ascribed to protein aggregation, since they were independent from protein concentration. In addition, note that the same elution profiles were obtained regardless of whether a sodium phosphate or Tris/HCl buffer were used for elution and irrespective of the NaCl concentration.

The insets of **Fig. 5** show the Stokes radii (R_S_^obs^) of *Henipavirus* PNT and N**_T_**_AIL_, as deduced from the apparent molecular mass observed in gel filtration [51]. By comparing each experimentally determined R_S_^obs^ with the theoretical Stokes radii expected for various conformational states (R_s_^NF^: monomeric natively folded protein; R_s_^Trim^: trimeric folded protein; R_s_^U^ : fully unfolded RC state in urea; R_s_^PMG^: PMG conformation) all the protein domains were found to have Stokes radii similar to the expected values of either PMG-like IDPs or of folded trimers (see insets in **Fig. 5**). Indeed, as seen in **Fig. 5**, the R_S_^obs^ of all the proteins are much larger than the corresponding R_s_^NF^ values, and are very close to the values expected either for a PMG or for a folded trimer (see ratios between R_S_^obs^ and R_S_^PMG^ or R_S_^Trim^ in insets of **Fig. 5**). In addition, the comparison of the hydrodynamic volume (V_h_^obs^) of each protein, as calculated from the R_S_^obs^, with the theoretical volume values expected for the various conformational states, points out a better agreement with the expected values of PMG-like IDPs than with those of folded trimers (see ratios between V_h_^obs^ and V_h_^PMG^ or V_h_^Trim^ in insets in **Fig. 5**).

These studies suggest that *Henipavirus* PNT and N_TAIL_ proteins either adopt a PMG conformation or are folded trimers.

### NMR studies of *Henipavirus* PNT and N_TAIL_

**Figure 6:**
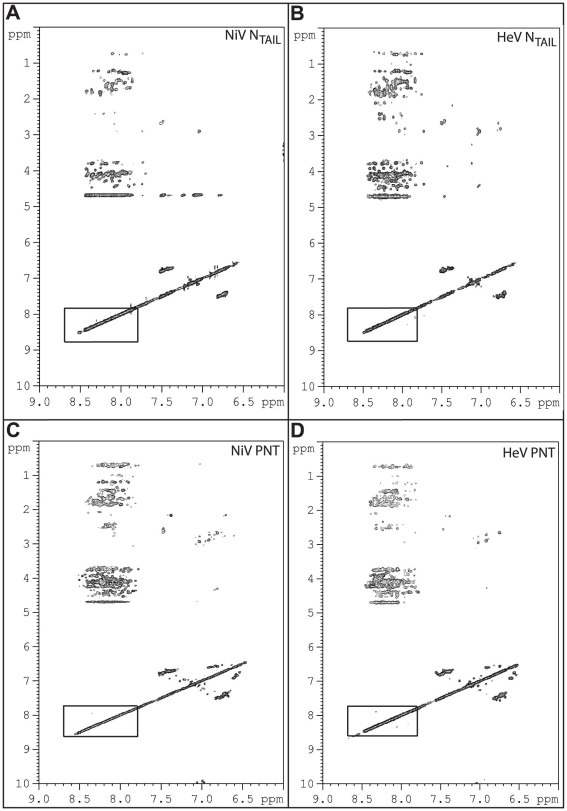
Two-dimensional ^1^H NMR NOESY spectra of *Henipavirus* N_TAIL_ and PNT proteins. Spectra were recorded at 300K with protein samples at 0.1 mM in 10 mM sodium phosphate pH 7, 150 mM NaCl and 10% D_2_O. *ppm*: values for resonance shifts in parts *per* million of the spectrophotometer frequency. The frames show the small spread of the resonance frequencies for amide protons.

In order to discriminate between these two latter hypotheses and to directly assess the actual conformation of *Henipavirus* PNT and N**_TAIL_** proteins, we studied them by 2D NMR spectroscopy. **Fig. 6** shows the amide region of their NOESY spectra. The very small spread of the resonance frequencies for amide protons (between 7.8 ppm and 8.7 ppm, see frames in **Fig. 6**) together with the scarcity of NOEs in the amide-amide region are typical of proteins without any stable secondary structure (for examples see [6], [18]), thereby supporting lack of a packed core within *Henipavirus* PNT and N_TAIL_ domains.

### CD studies of *Henipavirus* PNT and N_TAIL_

**Figure 7:**
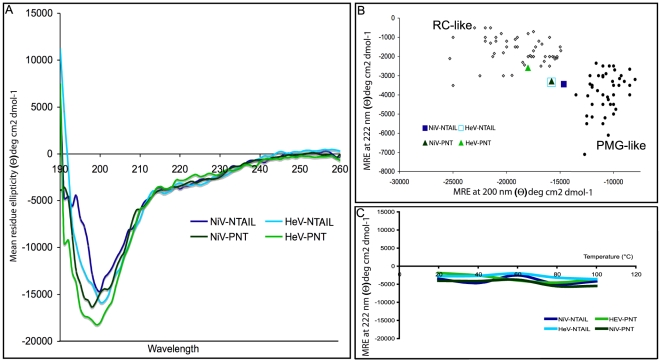
Analysis of *Henipavirus* N_TAIL_ and PNT proteins by far-UV circular dichroism. (**A**) Far-UV CD spectra of *Henipavirus* N_TAIL_ and PNT proteins at 0.1 mg/mL in 10 mM sodium phosphate pH 7 at 20°C. Data are representative of one out of three independent acquisitions. (**B**) 222-200 ellipticity plot modified from Uversky [32]. The mean residue ellipticity at 222 nm of a set of well-characterized unfolded or premolten globule proteins (from [32]) has been plotted against the mean residue ellipticity at 200 nm. The position in the plot of *Henipavirus* N_TAIL_ and PNT is highlighted. (**C**) Mean residue ellipticity at 222 nm as a function of the T. Data are representative of one out of three independent experiments. Protein and buffer concentrations were the same as described in (**A**).

In further support of the absence of an ordered structure, the far-UV CD spectra of *Henipavirus* PNT and N_TAIL_ at neutral pH are typical of unstructured proteins, as seen from their large negative ellipticity at 198 nm and low ellipticity at 190 nm (**Fig. 7A**). However, the observed ellipticity values at 200 and 222 nm of *Henipavirus* N**_TAIL_** and of NiV PNT are consistent with the existence of some residual secondary structure, as observed in IDPs adopting a PMG conformation (**Fig. 7B**). Indeed, Uversky noticed that IDPs can be subdivided in PMG-like and RC-like as a function of their ellipticity values at 200 and 222 nm [32]. Strikingly, except for HeV PNT that falls in the RC-like region of the plot, the other *Henipavirus* domains are all located in the twilight zone between RC-like and PMG-like proteins. This suggests that NiV PNT, as well as both *Henipavirus* N_TAIL_ domains, do not adopt a fully extended conformation, contrary to HeV PNT that has a tendency to be more flexible and possesses less residual structure.

We also monitored the ellipticity at 222 nm of *Henipavirus* PNT and N**_T_**_AIL_ proteins at increasing temperatures (**Fig. 7C**). In spite of the rather noisy (i.e. undulating) nature of the obtained curves, no *cooperative* thermal transitions were observed, as judged based on the lack of a coherent trend in the variation of the ellipticity at 222 nm as a function of the temperature (**Fig. 7C**). These results, once again, support lack of a rigid 3D structure [52].

**Figure 8:**
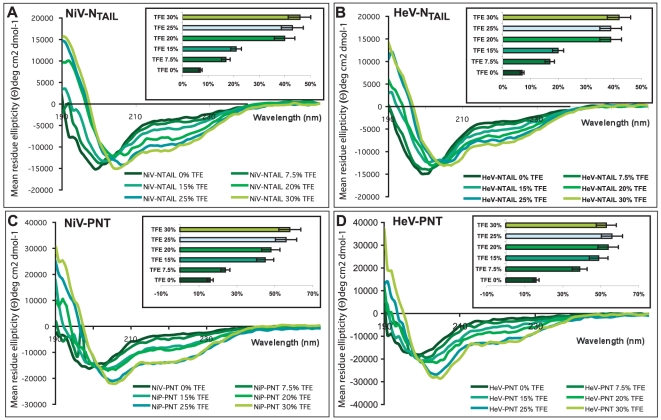
Far-UV CD spectra of *Henipavirus* Ntail and PNT proteins in TFE. Far-UV CD spectra of of *Henipavirus* N_TAIL_ and PNT proteins in10 mM sodium phosphate pH 7 at 20°C and in the presence of increasing concentrations of TFE (0, 7.5, 15, 20 and 30%). Protein concentrations ranged from 0.06 mg/mL (30% TFE) to 0.1 mg/mL (0% TFE). Data are representative of one out of three independent acquisitions. The insets show the α-helical content at the various TFE concentrations estimated by CDSSTR (see Materials and Methods). The error bar (10% of the value) corresponds to the experimentally determined standard deviation from three independent experiments.

To test the potential of *Henipavirus* PNT and N_TAIL_ folding, we recorded their CD spectra in the presence of increasing concentrations of TFE. The solvent TFE is widely used as an empirical probe of hidden structural propensities of peptides and proteins as it mimics the hydrophobic environment experienced by proteins in protein-protein interactions [53], [54], [55] (**Fig. 8**). All the proteins show an increasing gain of α-helicity upon addition of TFE, as indicated by the characteristic maximum at 190 nm and double minima at 208 and 222 nm (**Fig. 8**). The α-helical content gradually increases upon increasing the TFE concentration from 0 to 25% and then reaches a *plateau*, while no concomitant dose-dependent increase in the content of β structure is detected (data not shown).

Most unstructured-to-structured transitions take place in the presence of 20% TFE, a concentration at which the α-helix content is estimated to reach approximately 40% for both N_TAIL_ and 50% for PNT proteins (**Fig. 8**). Note that for all the proteins, the spectra display an isodichroic point at 202 nm indicative of a two-state transition (**Fig. 8**).

### Dynamic light scattering studies of *Henipavirus* PNT and N_TAIL_

In view of further investigating the extent of disorder within *Henipavirus* PNT and N_TAIL_ proteins, we carried out dynamic light scattering (DLS) studies in the presence or absence of urea. This approach has the advantage of allowing the Stokes radius to be directly measured, as opposite to SEC analyses that only provide an estimation of the Stokes radius.

These studies showed that all *Henipavirus* PNT and N_TAIL_ protein samples are highly monodisperse (99%), consisting of a single protein species. While the HeV and NiV N_TAIL_ proteins possess a very similar R_S_ (28±2 Å or 26±1 Å for NiV and HeV, respectively), the two PNT proteins differ in their R_S_, which was either 50±3 Å or 44±3 Å, depending on whether the HeV or NiV PNT protein was studied (see **Table 1**). For the N_TAIL_ and NiV PNT proteins, these values are consistent (within the error bars) with the R_s_ measured by SEC, while the R_s_ of HeV PNT was found to be slightly larger (see **Table 1**).

**Table 1:** Stokes radii as obtained by either SEC or DLS analyses in the presence or absence of urea.

	*R_S_ (SEC)*	*R_S_ (DLS)−Urea*	*R_S_ (DLS)* + *Urea*
NiV N_TAIL_	28±2 Å	28±2 Å	37±2 Å
HeV N_TAIL_	28±2 Å	26±1 Å	37±2 Å
NiV PNT	44±2 Å	44±3 Å	55±2 Å
HeV PNT	44±2 Å	50±3 Å	57±2 Å

The average values as obtained from three independent measurements are shown.

In view of highlighting possible denaturation-induced loss of compactness, we also carried out these measurements in the presence of urea. The obtained R_S_ values (37±2 Å for both N_TAIL_ proteins, and either 55±2 Å or 57±2 Å for NiV and HeV PNT, respectively) highlight a significant increase in the hydrodynamic radius in the presence of urea (see **Table 1**). These results argue for the presence of residual intramolecular interactions within *Henipavirus* PNT and N_TAIL_ proteins under native conditions, as expected for proteins adopting a PMG conformation.

## Discussion

### *Henipavirus* PNT and N_TAIL_ as members of the premolten globule sub-family

The peculiar sequence properties of *Henipavirus* PNT and N**_TAIL_** suggest that these protein domains are mostly unstructured in solution.

In agreement, they show an aberrant electrophoretic migration, which is a hallmark of protein disorder [46], and display a high protease-sensitivity that argues for the lack of a packed core in these protein domains. Likewise, CD studies with increasing temperatures showed lack of any cooperative thermal unfolding, thus supporting once again the lack of a packed core. Indeed, IDPs are rather insensitive to temperature increase, with some of them having even been reported to undergo heat-induced folding (see [47] and references therein cited, and [56], [57]).

The hydrodynamic properties of *Henipavirus* PNT and N**_TAIL_** inferred from gel filtration are consistent with these protein domains being either stable globular trimers, or extended (unstructured) monomers. Using NMR and CD we show that they are actually disordered in solution. However, with the only notable exception of HeV PNT, their far-UV spectroscopic parameters (see **Fig. 7B**) indicate that they are not fully unfolded, but rather they conserve some transiently populated secondary structure content typical of IDPs with a PMG conformation [32]. In addition, the mean hydrodynamic volumes and Stokes radii inferred from gel filtration are close to the values expected for native PMG conformations [32]. In further support of the occurrence of some residual structure, DLS studies pointed out a significant increase in the Stokes radius of all proteins upon addition of urea. The R_S_ values obtained in the presence of urea are close to those expected for fully extended forms (*cfr* **Table 1** and **Fig. 5**). The HeV PNT protein has a notable behavior in that its Stokes radius, as measured by DLS, is slightly, though significantly, larger than that inferred from SEC studies. This observation nicely correlates with the above-mentioned spectroscopic properties of HeV PNT as seen by far-UV CD studies (see **Fig. 7B**). With the only notable exception of HeV PNT, for all the other proteins herein studied, the R_S_ values provided by DLS were found to be very similar to those obtained by SEC. This confirms and extends previous thorough SEC analyses of several proteins revealing that the hydrodynamic radius values inferred from SEC are in very good agreement with those obtained by other hydrodynamic methods, such as viscometry, analytical ultracentrifugation and dynamic light scattering (DLS) (see [58] and references therein cited).

According to [32], *Henipavirus* PNT and N**_TAIL_** proteins lie in a region of the CH-plot that is consistent with the occurrence of residual intramolecular interactions typical of native PMGs. In particular, Uversky showed that intrinsic coils are more distant from the border separating structured proteins from IDPs, than intrinsic PMGs [32]. It should be pointed out however that a systematic experimental confirmation of the relationship between position in the CH-plot and content in secondary structure is still lacking, with a few discrepancies having even been experimentally observed [59]. The distance values from the border (H_boundary_−H) are 0.034 and 0.007 for HeV and NiV N**_TAIL_** respectively, whereas they are 0.050 and 0.031 for HeV and NiV PNT proteins, respectively (see Materials and Methods). According to [32], these values are all consistent with the values expected for native PMGs (0.037±0.033). Interestingly, although the distance value of HeV PNT is compatible with a PMG state, this protein domain has the largest distance from the border separating IDPs and structured proteins (see **Fig. 3**). This finding is in agreement with the spectroscopic parameters of HeV PNT that locate it in the proximity of RC-like proteins (see **Fig. 7B**). Notably, HeV N**_TAIL_** and NiV PNT, which fall in the same position of the ellipticity plot (see **Fig. 7B**), display comparable distances from the boundary of the CH-plot (see **Fig. 3**).

Thus, *Henipavirus* PNT and N**_TAIL_** can be described as non-globular polypeptide chains, more compact than RCs, all containing some residual structure. This residual structure restrains the conformational space sampled by these proteins, thereby reducing the number of interconverting conformers in solution. In agreement, the distribution of the conformations of *Henipavirus* PNT and N**_TAIL_** is narrow, as seen by the relative sharpness of the elution peaks observed in gel filtration (see **Fig. 5**).

### Residual structure and folding propensities of *Henipavirus* PNT and N_TAIL_

Analysis of the HCA plot of *Henipavirus* N proteins, reveals the presence within N_TAIL_ of four short regions possessing each a hydrophobic cluster and/or a predicted (α or β) secondary structure elements , are found (see **Fig. 1A**). These short order-prone segments might correspond to Molecular Recognition Elements (MoREs), where these latter are short order-prone regions within IDRs with a propensity to undergo induced folding (i.e. a disorder-to-order transition) upon binding to a partner. MoREs can be divided in α-, β- and irregular (i.e. neither α, nor β) MoRES depending on the nature of the structural transition they undergo [60], [61], [62], [63]. Based on the type of predicted secondary structure element, *Henipavirus* N_T**AIL**_ domains may possess two putative α-MoREs (residues 408–422 and 473–493), a putative I-MoRE (residues 523–532) and a putative β-MoRE (residues 444–464). Thus, the residual ordered secondary structure present within *Henipavirus* PNT and N**_TAIL_** likely arises from transiently populated MoREs. That the conformational space sampled by these interaction-prone short segments within IDRs can be restricted even in the absence of the partner has already been reported [64] and assumed to reflect an inherent conformational preference. It has been proposed that these partly pre-configured MoREs can enable a more efficient start of the folding process induced by a binding partner through a reduction of the entropic cost of binding [46], [61], [62], [64], [65], [66].

The solvent TFE mimics the hydrophobic environment experienced by proteins in protein-protein interactions and is therefore widely used as a probe to unveil the propensity of IDPs to undergo induced folding upon target binding [54]. In agreement with the presence of transiently populated α-helical segments, CD studies in the presence of TFE pointed out a clear α-helical potential in *Henipavirus* PNT and N**_TAIL_**. Note that the gain of α-helicity induced by TFE, although already reported for other proteins, including MeV PNT [18] and N**_TAIL_** [6], [19], is not a general rule: for instance, *(i)* the acidic activator domain of GCN4 forms little or no α-helix in TFE concentrations as high as 30% and folds mostly as β-sheets in 50% TFE [67] and *(ii)* the intrinsically disordered rat seminal vesicle protein IV exclusively undergoes β transitions in the presence of TFE (P. Palladino, S. Vilasi, R. Ragone and F. Rossi, personal communication). Furthermore, in the case of MeV N**_TAIL_**, we have recently shown that TFE promotes α-helical folding of the 488–502 region only, with the downstream region only becoming slightly less mobile while retaining an extended conformation in the presence of 20% TFE [68]. The α-helical propensity of *Henipavirus* PNT and N**_TAIL_** is in agreement with the occurrence of two putative α-MoREs within both N**_TAIL_** proteins, as well as with the presence of a 50-residues long α-helical region at the N-terminus of both PNT domains (see **Fig. 1**). On the other hand, secondary structure predictions point to the presence of a short β-strand within the second putative MoRE of both NiV (*aa* 444–452) and HeV (aa 444–449) N**_TAIL_** (see **Fig. 1A**), thereby suggesting that this MoRE could be a β-MoRE. However, based on the experimentally observed behavior of both N_TAIL_ domains in the presence of TFE, this latter MoRE is probably α-helical one. This is consistent with the shape and size of hydrophobic clusters occurring in this region, as seen from the HCA plot (see **Fig. 1A**) [69] and is also in agreement with previous experimental observations that showed the helical nature of the cognate MeV and SeV N**_TAIL_** MoREs [19], [20], [42], [68], [70], [71], [72], [73]. Definite answers as to the involvement of these putative MoREs in binding and in structural transitions await identification of partner(s), as well as generation of truncated N**_TAIL_** constructs and assessment of their binding abilities. By analogy with MeV and SeV, we can speculate that one such a possible binding partner could be the X domain of the P protein [19], [20], [25], [42], [68], [70], [71], [72], [73]. Future studies will address the ability of *Henipavirus* N_TAIL_ domains to effectively interact with XD, and will assess possible inter-species cross-interactions, as well as possible structural transitions within N_TAIL_ upon interaction with XD.

### Structural disorder as a lessener of evolutionary constraints

Notably, PNT partially overlaps with the C protein (being encoded by the same RNA region) and the spacer region partially overlaps with the C-terminal domain of the V protein. The disordered nature of PNT and of the “spacer” region connecting PNT to PMD likely reflects a way of alleviating evolutionary constraints within overlapping reading frames. This observation is in agreement with previous reports pointing out a relationship between overlapping genes and structural disorder [26], [74], [75], [76], [77], as also nicely illustrated by recent findings showing that the hepatitis C virus Core +1/S protein, overlapping the Core protein gene in the +1 reading frame, is intrinsically disordered [78]. Disorder, which is encoded by a much wider portion of sequence space as compared to order, can indeed represent a strategy by which genes encoding overlapping reading frames can lessen evolutionary constraints imposed on their sequence by the overlap, allowing the encoded overlapping protein products to sample a wider sequence space without losing function.

By comparing the modular organization of the P proteins within the *Paramyxovirinae* subfamily, we noticed that a larger PNT domain in *Henipavirus*es accounts for the extra length of their P protein (*cfr.* **Fig. 1B** with Fig. 8 in [26]). This finding is consistent with the higher tolerance of disordered regions to insertions or major rearrangements as compared to ordered ones.

### Functional advantage of disorder within *Henipavirus* N and P proteins

The results herein presented clearly show that intrinsic disorder is abundant within the replicative complex of *Henipaviruses*, in agreement with our previous findings for other *Paramyxovirinae* members (see [26] and references therein cited). One of the functional advantages of disorder is related to an increased plasticity that enables IDRs to bind to numerous structurally distinct targets [79], [80], [81] and allows protein interactions to occur with both high specificity and low affinity [27], [30], [31], [34], [82], [83], [84], [85], [86]. In the case of MeV, the N**_TAIL_** domain has indeed been shown to bind to numerous partners including the X domain of the P protein [6], [19], [20], [42], the major inducible heat shock protein hsp70 [87], [88], [89], the interferon regulatory factor 3 [90], [91], a yet unidentified protein cell receptor involved in MeV-induced immunosuppression [92], [93], a nuclear export protein [94], the matrix protein [95] and, possibly, components of the cell cytoskeleton [96], [97]. Likewise, both MeV and SeV PNT domains have been reported to interact with multiple partners, with the former interacting with N [98] and cellular proteins [99], and the latter interacting with the unassembled form of N (N°) and the L protein [100], [101]. By analogy with the Sendai [100], [101] and human parainfluenza virus type 2 [102], the N-terminal α-helical segment within *Henipavirus* PNT could correspond to the N°-binding region. Given its relative shortness and positioning upstream a disordered region, this N-terminal α-helical segment is not probably stably folded in isolation and rather only folds in cooperation with another protein. Although we can't formally rule out the possibility that *Henipavirus* PNT and N**_TAIL_** domains may undergo some degree of folding in the context of the full-length P and N proteins, a few studies carried out on the cognate domains of related viruses suggest that the *Henipavirus* PNT and N**_TAIL_** domains could display a significant amount of disorder within the entire P and N proteins either. Indeed, in SeV, the PNT region has been shown to be disordered not only in isolation but also in the context of the full-length protein [103], [104], as were the linker region between PMD and XD in the context of PCT [105] and the region upstream XD within the PX protein [106], [107]. Likewise, the MeV N**_TAIL_** region is also disordered within the entire nucleoprotein, being equally accessible to monoclonal antibodies in isolation and within recombinant nucleocapsids, being highly susceptible to protease digestion and not visible in electron microscopy [6]. Definite answers as to the disordered state of PNT and N_TAIL_ domains in the context of the full-length N and P proteins require however additional experimental work that will be the focus of future studies.

Likewise, as for the possibility that PNT and N**_TAIL_** are effectively disordered *in vivo*, further experimental work is required to address this question. In this regard, it is noteworthy that, so far, available data addressing the disordered state of IDPs *in vivo* are controversial: if in-cell NMR experiments on the natively unfolded FlgM protein indeed suggest a more folded conformation in the cellular environment of live bacteria [108], a few reports support a disordered state for other IDPs either in living cells or in the presence of crowding agents that mimic the crowded environment of cells (for examples see [6], [109], [110]).

### Abundance of structural disorder within viruses

A recent study showed that viral proteins, and in particular RNA virus proteins, are enriched in disordered regions [111]. In that study, the authors propose that beyond affording a broad partnership, the wide occurrence of disordered regions in viral proteins could also be related to the typical high mutation rates of RNA viruses, representing a strategy for buffering the deleterious effects of mutations [111]. Taking into account these considerations, as well as the correlation between overlapping genes and disorder [74], [75], [76], we propose that the main advantage of the abundance of disorder within viruses would reside in pleiotropy and genetic compaction. Indeed, disorder provides a solution to reduce both genome size and molecular crowding, where a single gene would *(i)* encode a single (regulatory) protein product that can establish multiple interactions via its disordered regions and hence exert multiple concomitant biological effects, and/or *(ii)* would encode more than one product by means of overlapping reading frames. In fact, since disordered regions are less sensitive to structural constraints than ordered ones, the occurrence of disorder within one or both protein products encoded by an overlapping reading frame can represent a strategy to alleviate evolutionary constraints imposed by the overlap. As such, disorder would confer to viruses the ability to “handle” overlaps, thus further expanding the coding potential of viral genomes.

### Conclusions

This paper represents the first report on the experimental characterization of *Henipavirus* N and P proteins. It also provides new perspectives in the study of disordered regions within the replicative complex of these viruses. In particular, taking into account the finding that protein-protein interactions mediated by disordered regions have been proved to be interesting drug discovery targets with the potential to increase significantly the discovery rate for new compounds [112], the present results designate the disordered regions of *Henipavirus* N and P proteins as promising targets for antiviral compounds. In addition, the broad molecular partnership that typifies IDRs, suggests that *Henipavirus* PNT and N_TAIL_ domains could be involved in manifold protein-protein interactions. As such, the results herein presented could orient future work towards the identification of both viral and cellular partners. In the long term, studies focused on the interactions with binding partners are expected to contribute to shed light on the molecular mechanisms of the N and P proteins of these highly pathogenic agents. On a last note, the present study is also expected to contribute to future efforts aimed at obtaining high-resolution structural data on *Henipavirus* N and P proteins by helping to delineate domains of these latter amenable to crystallization.

## Materials and Methods

### Sequence retrieval and disorder prediction

Sequences for this study were obtained from the VaZyMolO data base [40]. Sequence accession numbers for N are VaZy82 (HeV) and VaZy1 (NiV). Sequence accession numbers for P are VaZy83 (HeV) and VaZy2 (NiV). Sequence similarity and identity were calculated using the Emboss program http://www.ebi.ac.uk/Tools/emboss/align/index.html. Multiple sequence alignment of *Henipavirus*, *Morbillivirus* and *Respirovirus* N proteins were obtained using ClustalW [113] (http://www.ebi.ac.uk/Tools/clustalw2/index.html) and drawn using ESPript [114] (http://espript.ibcp.fr/ESPript/cgi-bin/ESPript.cgi). Secondary structure predictions were carried out using the PSIPRED sever [115] (http://bioinf.cs.ucl.ac.uk/psipred/).

Predictions of disorder were carried out using the Metaserver of Disorder MeDor, which is freely available from http://www.vazymolo.org/MeDor/index.html. MeDor collects disorder and secondary structure predictions from servers available on the web and generates a graphical output [36]. Specifically, it uses predictions from 10 disorder predictors, namely IUPred [116], Prelink [117], RONN [118], FoldUnfold [119], [120], DisEMBL [121], Foldindex [122], Globplot2 [123], Disprot VL3, Disprot VL3H [124], Disprot VSL2B [125], and performs secondary structure prediction using the pred2ary algorithm using the default parameters [126]. It also incorporates hydrophobic cluster analysis (HCA) [69] and generates a HCA plot. Since MeDor generates no automated consensus on disorder, the assignment of order and disorder was done taking into account the length of the concerned region and the accuracy of the various predictors in identifying regions of short (<30 residues) or long (>70 residues) disorder (for reviews on the identification of disorder see [41], [127], [128], [129]. Coiled-coils, which correspond to regions that often fool some predictors into giving wrong predictions (see [41], [127] and references therein cited), were first identified by visual inspection of the HCA plot and then confirmed using the COILS (http://www.ch.embnet.org/software/COILS_form.html) [130] and 3D-PSSM (http://www.sbg.bio.ic.ac.uk/~3dpssm/index2.html) [131] servers.

Small hydrophobic clusters occurring within mainly disordered regions, as observed in HCA plots, were assumed to correspond to putative Molecular Recognition Elements (MoREs), where these latter are short order-prone regions within IDRs that fold upon binding to a partner or ligand [60], [61], [62], [63].

### *In silico* amino acid composition analysis

Deviations in amino acid composition of *Henipavirus* PNT and N_TAIL_ were computed as already described [30], [132], using the average amino acid frequencies of the SWISS-PROT database (as obtained from http://us.expasy.org/sprot) as the reference value. The average amino acid frequencies of the SWISS-PROT database roughly corresponds to the mean composition of proteins in nature. If the average composition of an amino acid X in SWISS-PROT proteins is CW_X_, and CP_X_ is the composition of X within a protein P, deviation from the composition of X of SWISS-PROT proteins was defined for P as (CP_X_-CW_X_)/CW_X_.

Low sequence complexity segments were searched using the SEG server (http://mendel.imp.ac.at/METHODS/seg.server.html) [133] with a trigger window length of 25, a trigger complexity of 2.2 and an extension complexity of 2.5 that work well for the identification of short non-globular domains.

### Charge-Hydropathy (CH) plots

Charge-hydropathy (CH) plots were then generated as described by Uversky et al. [32]. The CH plot is divided into two regions by a line, which corresponds to the equation H = (R+1.151)/2.785, where R is the mean net charge and H is the mean hydrophobicity. In the left part of the diagram (where H<(R+1.151)/2.785), a protein is predicted as disordered, whereas it is predicted as ordered in the right part [32]. H_Boundary_ was computed according to [32]: H_Boundary_ = (R+1.15)/2.785. The mean net charge (R) of a protein is defined as the absolute value of the difference between the number of positively and negatively charged residues at pH 7 divided by the total number of amino acid residues. It was calculated using the program ProtParam at the EXPASY server (http://www.expasy.ch/tools). The mean hydrophobicity (H) is the sum of normalized hydrophobicities of individual residues divided by the total number of amino acid residues minus 4 residues (to take into account fringe effects in the calculation of hydrophobicity). Individual hydrophobicities were determined using the Protscale program at the EXPASY server (http://www.expasy.ch/tools), using the options “Hphob/Kyte & Doolittle”, a window size of 5, and normalizing the scale from 0 to 1. The values computed for individual residues were then exported to a spreadsheet, summed and divided by the total number of residues minus 4 to yield (H). The net charge-hydrophobicity method is only applicable to a protein (or protein region) provided it is not composed of shorter, structurally independent modules. It might otherwise give conflicting results. It was only validated for regions >50 aa [45]. An estimation of its error rate can be drawn from Uversky [50]. In that study, no globular protein was found to have a ratio located on the left side of the line, indicating that the positive error rate for the prediction of disordered proteins must be very low. However, five unfolded proteins out of 105 – which were all borderline – were wrongly assigned as being globular, indicating a negative error rate of about 5%.

### Construction of expression plasmids

The *Henipavirus* N_TAIL_ and PNT constructs, encoding residues 400–532 of N and 1–406 (NiV) or 1–404 (HeV) of P, with a hexahistidine tag fused to their N- (N_TAIL_) or C-termini (PNT), have been obtained by PCR using *Pfx* polymerase (Stratagene) and synthetic N and P genes (GenScript), optimized for the expression in *E. coli*, as templates. Primers (Operon) were designed to introduce a hexahistidine tag encoding sequence either at the 5′ (N_TAIL_) or 3′ (PNT) end of the DNA fragments, as well as an *AttB1* and *AttB2* sites at the 5′ and 3′ ends of these latter, respectively. The rationale for the choice of the tag position was to reflect at best the “natural” organization of the proteins, where additional (natural) residues are found upstream N_TAIL_ and downstream PNT. After purification (PCR Purification Kit, Qiagen), the PCR products were cloned into the pDest14 vector (Invitrogen) using the Gateway recombination system (Invitrogen).

Selection and amplification of DNA constructs was carried out using CaCl2-competent *E. coli* TAM1 cells (Active Motif). The sequence of the coding region of all expression plasmids was verified by sequencing (GenomeExpress).

### Bacterial expression of *Henipavirus* N_TAIL_ and PNT constructs

The *E. coli* Rosetta [DE3] pLysS strain (Novagen) was used for the expression of the constructs. This strain, which is optimized for the expression of recombinant proteins, also carries the lysozyme gene thus allowing a tight regulation of the expression of the recombinant gene, as well as a facilitated lysis. Cultures were grown overnight to saturation in LB medium containing 100 µg/ml ampicilin and 34 µg/ml chloramphenicol. An aliquot of the overnight culture was diluted 1/25 in LB medium and grown at 37°C. At OD_600_ of 0.7, isopropyl ß-D-thiogalactopyranoside (IPTG) was added to a final concentration of 0.2 mM, and the cells were grown at 37°C for 3 hours. The induced cells were harvested, washed and collected by centrifugation. The resulting pellets were frozen at −20°C.

### Purification of *Henipavirus* N_TAIL_ and PNT

All cellular pellets, irrespective of the recombinant protein they express, were resuspended in 5 volumes (v/w) buffer A (50 mM sodium phosphate pH 7, 300 mM NaCl, 10 mM Imidazole, 1 mM phenyl-methyl-sulphonyl-fluoride (PMSF)) supplemented with lysozyme 0.1 mg/mL, DNAse I 10 µg/mL, protease inhibitor cocktail (Roche) (one tablet for 50 mL of bacterial lysate). After a 20 min incubation with gentle agitation, the cells were disrupted by sonication (using a 750 W sonicator and 4 cycles of 30 s each at 45% power output). The lysate was clarified by centrifugation at 30,000 g for 30 min. Starting from a 1 L culture, the clarified supernatant was incubated for 1 hr with gentle shaking with 4 mL Chelating Sepharose Fast Flow Resin preloaded with Ni^2+^ ions (GE, Healthcare), previously equilibrated in buffer A. The resin was washed with buffer A containing 20 mM imidazole, and the recombinant protein was eluted in buffer A containing 250 mM imidazole. Eluates were analyzed by SDS-PAGE for the presence of the desired protein product. The fractions containing the recombinant protein were combined, and then loaded onto a Superdex 200 HR 16/60 column (GE, Healthcare). N_TAIL_ proteins were eluted in either 10 mM Tris/HCl pH 8, 500 mM NaCl or 10 mM sodium phosphate pH 7, 150 mM NaCl depending on whether the protein was further subjected to limited proteolysis or to NMR and CD analyses, respectively. Likewise, PNT proteins were either eluted in 10 mM Tris buffer pH 8 containing 300 mM NaCl or in 10 mM sodium phosphate pH 7, 150 mM NaCl. For both HeV and NiV N_TAIL_ proteins, which are devoid of Trp and Tyr residues, the elution was followed by monitoring the absorbance at 254 nm instead of 280 nm.

The proteins were concentrated using Centricon Plus-20 (molecular cutoff of either 5,000 Da or 10,000 Da for N_TAIL_ or PNT proteins, respectively) (Millipore). All proteins were stored at −20°C either in the presence (PNT) or absence (N_TAIL_) of 10% glycerol. HiTrap desalting columns (5 mL) (GE, Healthcare) where used to get rid of glycerol prior to NMR experiments, as well as to reduce the NaCl concentration in view of CD studies. All purification steps, except for gel filtrations, were carried out at 4°C.

### Hydrodynamic characterization of *Henipavirus* PNT and N_TAIL_

Apparent molecular mass (MMapp) of proteins eluted from gel filtration columns was deduced from a calibration carried out with LMW calibration kits (GE, Healthcare). The hydrodynamic radius of a protein (Stokes radius) can be deduced from its apparent molecular mass (as seen by gel filtration) [51]. The theoretical Stokes radii (R_s_) (in Å) of a natively folded (R_s_^NF^), fully unfolded RC state in urea (R_s_^U^) and natively unfolded PMG (R_s_^PMG^) protein with a theoretical molecular mass (MMtheo) (in Daltons) were calculated according to [50]:
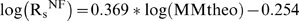
(1)

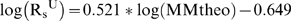
(2)

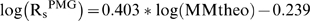
(3)The theoretical Stokes radii (R_s_) of a natively folded trimer (R_s_^Trim^) was calculated as:
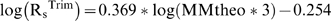
(4)


The hydrodynamic volume (V_h_) was calculated from the experimentally observed Stokes radius as V_h_ = 4/3 π R_s_^3^. The theoretical hydrodynamic volumes of a native (V_h_^NF^), fully unfolded (V_h_^U^) and natively unfolded PMG (V_h_^PMG^) protein of N residues, were calculated according to [32]:
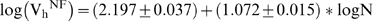
(5)

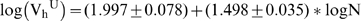
(6)

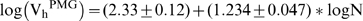
(7)


According to [32], the expected hydrodynamic volume of a natively folded trimer (V_h_^Trim^) was calculated as:
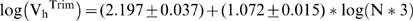
(8)


### Determination of protein concentration

Protein concentrations were calculated either using the theoretical absorption coefficients ε (mg/ml.cm) at 280 nm as obtained using the program ProtParam at the EXPASY server (http://www.expasy.ch/tools) (PNT proteins), or the BCA protein assay reagent (Pierce) (N_TAIL_ proteins).

### Digestion of *Henipavirus* N_TAIL_ and PNT by thermolysin

A thermolysin stock solution was prepared by dissolving the commercial powder (Sigma, 50–100 units/mg protein) at a concentration of 0.4 mg/mL in 10 mM Tris/HCl pH 8, 300 mM NaCl and then stored at −20°C. PNT and N_TAIL_ samples at a concentration of 2.5 mg/mL in 10 mM Tris/HCl pH 8 supplemented with either 300 mM (PNT) or 500 mM (N_TAIL_) NaCl, were used as stock solutions. Lysozyme (Euromedex) was used as a control. Digestions of proteins were performed by incubation of the protein substrate (1 mg/mL) with thermolysin in 20 mM Tris/HCl pH 8 at 26°C. Protease∶protein substrate ratios were 1∶100 (w/w). The extent of proteolysis was evaluated by SDS–PAGE analysis of 10 µl aliquots removed from the reaction mixture over a time course (0, 1 and 24 hours), added to 10 µl of 2× Laemli sample buffer and boiled for 5 min to inactivate the protease.

### Mass Spectrometry (MALDI-TOF)

Mass analysis of *Henipavirus* N_TAIL_ and PNT proteins was performed using an Autoflex II TOF/TOF. Spectra were acquired in the linear mode. Samples (0.7 µL containing 15 pmol) were mixed with an equal volume of sinapinic acid matrix solution, spotted on the target, then dried at room temperature for 10 min. The mass standard was either myoglobin or BSA depending on whether N_TAIL_ or PNT proteins were analyzed, respectively. Proteins were analyzed in the Autoflex matrix-assisted laser desorption ionization/time of flight (Bruker Daltonics, Bremen, Germany).

The identity of purified *Henipavirus* N_TAIL_ and PNT proteins was confirmed by mass spectral analysis of tryptic fragments. The latter was obtained by digesting (0.25 µg trypsin) 1 µg of purified recombinant protein obtained after separation onto SDS-PAGE. The tryptic peptides were analyzed as described above and peptide fingerprints were obtained and compared with *in-silico* protein digest (Biotools, Bruker Daltonics, Germany). The mass standards were either autolytic tryptic peptides or peptide standards (Bruker Daltonics).

### Two-dimensional Nuclear Magnetic Resonance (NMR)

PNT and N_TAIL_ samples at a concentration of 0.1 mM in 10 mM sodium phosphate pH 7, 150 mM NaCl and 10% D_2_O were used for the acquisition of a NOESY spectrum on a 600-MHz ultra-shielded-plus Avance-III Bruker spectrometer equipped with a TCI cryo-probe. The temperature was set to 300 K and the spectra were recorded with 2048 complex points in the directly acquired dimension and 512 points in the indirectly detected dimension. Solvent suppression was achieved by using excitations sculping with gradients [134]. The data were processed using the Bruker Topspin software; they were multiplied by a sine-squared bell and zero-filled to 1 K in first dimension prior to Fourier transformation.

### Circular Dichroism (CD)

CD spectra were recorded on a Jasco 810 dichrograph, equipped with a Peltier thermoregulation system, using 1-mm thick quartz cells in 10 mM sodium phosphate pH 7 at 20°C. CD spectra were measured between 190 and 260 nm, with a scanning speed of 20 nm/min and a data pitch of 0.2 nm. Spectra were averaged from three scans. Moreover, for each protein sample, at least three independent acquisitions were carried out so as to estimate the experimental error arising from sample preparation. The contribution of buffer was subtracted from experimental spectra. Spectra were smoothed using the “means-movement” smoothing procedure implemented in the SpectraManager package. Structural variations of both N_TAIL_ and PNT proteins were measured as a function of changes in the initial CD spectrum upon addition of increasing concentrations of 2,2,2-trifluoroethanol (TFE) (Fluka). The final NaCl concentration in PNT and N_TAIL_ samples was comprised between 5 and 15 mM.

Mean ellipticity values per residue ([Θ]) were calculated as [Θ] = 3300 m ΔA/(l c n), where l (path length) = 0.1 cm, n = number of residues, m = molecular mass in daltons and c = protein concentration expressed in mg/mL. Number of residues (n) are 140 for both HeV and NiV N_TAIL_, 410 for HeV PNT and 412 for NiV PNT, while m values are 15,241 Da for HeV N_TAIL_, 14,949 Da for NiV N_TAIL_, 45,216 Da for HeV PNT and 45,330 Da for NiV PNT. Protein concentrations ranged from 0.1 mg/mL (0% TFE) to 0.06 mg/mL (30% TFE). The experimental data in the 190–260 nm range were analyzed using the DICHROWEB website (http://dichroweb.cryst.bbk.ac.uk/html/home.shtml) which was supported by grants to the BBSRC Centre for Protein and Membrane Structure and Dynamics (CPMSD) [135], [136]. The CDSSTR deconvolution method was used to estimate the α-helical content using the reference protein set 7. Reconstructed curves very well superimposed on the experimental ones thus attesting the reliability of the inferred α-helical percentages (data not shown).

Measurements at fixed wavelength (222 nm) were performed in the temperature range of 20°C–100°C with data pitch 20°C and temperature slope of 5°C/min with protein concentrations of 0.1 mg/mL. The buffer solutions without the proteins were used as blanks.

### Dynamic light scattering studies of *Henipavirus* PNT and N_TAIL_

Dynamic light scattering experiments were performed with a Zetasizer Nano-S (Malvern) at 25°C. Protein samples were diluted in 10 mM Tris/HCl pH 8 in the presence or absence of urea to a final concentration of 0.5 mg/ml. The urea concentration ranged from 6.4 M to 7.4 M for PNT and N_TAIL_ proteins, respectively. The samples were filtered prior to the measurements (Millex syringe filters 0.22 µm, Millipore). The hydrodynamic radius was deduced from translational diffusion coefficients using the Stokes-Einstein equation. Diffusion coefficients were inferred from the analysis of the decay of the scattered intensity autocorrelation function. All calculations were performed using the software provided by the manufacturer. The relative viscosity of the samples containing 6.4 M or 7.4 M urea was assumed to be either 1.45 or 1.57, respectively, according to [137].

## Supporting Information

Figure S1 Multiple sequence alignment of *Henipavirus*, *Morbillivirus* and *Respirovirus* N proteins as obtained using ClustalW [113] (http://www.ebi.ac.uk/Tools/clustalw2/index.html) and ESPript [114] (http://espript.ibcp.fr/ESPript/cgi-bin/ESPript.cgi). Residues corresponding to a similarity above 60% are boxed and shown in red. Identical residues are boxed and shown in white on a red background. The front numbers correspond to the amino acid position in sequence. Dots above the alignment indicate intervals of 10 residues. Predicted secondary structure elements, as obtained using the PSIPRED server [115] (http://bioinf.cs.ucl.ac.uk/psipred/), for Hendra and Measles virus N are shown above the multiple sequence alignment. The red helix spanning residues 487–503 of Measles virus N corresponds to the helical segment observed in the crystal structure of a chimeric construct consisting of the C-terminal domain of the Measles virus P protein and of residues 486–504 of N (pdb code : 1T6O). The accession numbers of the N proteins are: NP 047106.1 (Hendra virus), NP 112021.1 (Nipah virus), Q89933.1 (measles virus), ABM64790.1 (Rinderpest virus), ABY61984.1 (Peste des Petits Ruminants virus), BAI60055.1 (Canine Distemper virus), AAB06278.1 (Sendai virus). A significant sequence divergence can be observed starting from position 400 up to the C-terminus.
(0.99 MB DOC)

Figure S2 Mass spectrometry (MALDI-TOF) analysis of recombinant, hexahistidine tagged NiV NTAIL purified from the soluble fraction of *E. coli*. Mass analysis was performed using an Autoflex II TOF/TOF. Spectra were acquired in the linear mode. The sample (0.7 µL containing 15 pmol) was mixed with an equal volume of sinapinic acid matrix solution, spotted on the target, then dried at room temperature for 10 min. The mass standard was myoglobin. Proteins were analyzed in the Autoflex matrix-assisted laser desorption ionization/time of flight (Bruker Daltonics, Bremen, Germany). A major peak with a mass slightly higher (14 953 Da) than expected (14 949 Da) was observed. The additional peak of 7 475 Da in mass, very probably corresponds to a degradation product, as the protein was shown to contain no contaminating proteins (as shown by mass spectrometry analysis of trypic fragments).
(0.05 MB DOC)

Figure S3 Mass spectrometry (MALDI-TOF) analysis of recombinant, hexahistidine tagged HeV NTAIL purified from the soluble fraction of *E. coli*. Mass analysis was performed using an Autoflex II TOF/TOF. Spectra were acquired in the linear mode. The sample (0.7 µL containing 15 pmol) was mixed with an equal volume of sinapinic acid matrix solution, spotted on the target, then dried at room temperature for 10 min. The mass standard was myoglobin. Proteins were analyzed in the Autoflex matrix-assisted laser desorption ionization/time of flight (Bruker Daltonics, Bremen, Germany). A peak with a mass slightly higher (15 304 Da) than expected (15 241 Da) was observed. The numerous additional peaks corresponding to species of lower molecular mass likely correspond to degradation products, as the protein was found to be devoid of contaminating protein by mass spectrometry analysis of tryptic fragments.
(0.06 MB DOC)

Figure S4 Mass spectrometry (MALDI-TOF) analysis of recombinant, hexahistidine tagged NiV PNT purified from the soluble fraction of *E. coli*. Mass analysis was performed using an Autoflex II TOF/TOF. Spectra were acquired in the linear mode. The sample (0.7 µL containing 15 pmol) was mixed with an equal volume of sinapinic acid matrix solution, spotted on the target, then dried at room temperature for 10 min. The mass standard was BSA. Proteins were analyzed in the Autoflex matrix-assisted laser desorption ionization/time of flight (Bruker Daltonics, Bremen, Germany). A major peak with a mass slightly higher (45 440 Da) than expected (45 330 Da) was observed. The additional peak (22 696 Da) very probably corresponds to a degradation product, as the protein was found to contain no contaminating proteins (as judged based by mass spectrometry analysis of tryptic fragments).
(0.06 MB DOC)

Figure S5 Mass spectrometry (MALDI-TOF) analysis of recombinant, hexahistidine tagged HeV PNT purified from the soluble fraction of *E. coli*. Mass analysis was performed using an Autoflex II TOF/TOF. Spectra were acquired in the linear mode. The sample (0.7 µL containing 15 pmol) was mixed with an equal volume of sinapinic acid matrix solution, spotted on the target, then dried at room temperature for 10 min. The mass standard was BSA. Proteins were analyzed in the Autoflex matrix-assisted laser desorption ionization/time of flight (Bruker Daltonics, Bremen, Germany). A major peak with a mass is slightly higher (45 342 Da) than expected (45 216 Da) was observed. The additional peak (22626 Da) very probably corresponds to a degradation product, as the protein was found to contain no contaminating proteins (as judged based by mass spectrometry analysis of tryptic fragments).
(0.06 MB DOC)

Figure S6 15% SDS-PAGE analysis of lysozyme after a 24 hours digestion by thermolysin. The digestion was performed by incubating lysozyme (1 mg/mL) with thermolysin in 20 mM Tris/HCl pH 8 at 26°C. The protease∶protein substrate ratio was 1∶100 (w/w). M: molecular markers. No degradation was observed even after an incubation period as long as 24 hours, consistent with the foldedness of the protein.
(0.07 MB DOC)

## References

[1] Eaton BT, Mackenzie JS, Wang LF, Fields BN, Knipe DM, Howley PM (2007). Henipaviruses.. Fields Virology. 5th ed.

[2] Drexler JF, Corman VM, Gloza-Rausch F, Seebens A, Annan A (2009). Henipavirus RNA in African bats.. PLoS ONE.

[3] Wang LF, Yu M, Hansson E, Pritchard LI, Shiell B (2000). The exceptionally large genome of Hendra virus: support for creation of a new genus within the family Paramyxoviridae.. J Virol.

[4] Eaton BT, Broder CC, Middleton D, Wang LF (2006). Hendra and Nipah viruses: different and dangerous.. Nat Rev Microbiol.

[5] Karlin D, Longhi S, Canard B (2002). Substitution of two residues in the measles virus nucleoprotein results in an impaired self-association.. Virology.

[6] Longhi S, Receveur-Brechot V, Karlin D, Johansson K, Darbon H (2003). The C-terminal domain of the measles virus nucleoprotein is intrinsically disordered and folds upon binding to the C-terminal moiety of the phosphoprotein.. J Biol Chem.

[7] Schoehn G, Mavrakis M, Albertini A, Wade R, Hoenger A (2004). The 12 A structure of trypsin-treated measles virus N-RNA.. J Mol Biol.

[8] Bhella D, Longhi S (2007). Measles virus nucleocapsid structure, conformational flexibility and the rule of six.. Measles virus nucleoprotein.

[9] Bhella D, Ralph A, Murphy LB, Yeo RP (2002). Significant differences in nucleocapsid morphology within the Paramyxoviridae.. J Gen Virol.

[10] Bhella D, Ralph A, Yeo RP (2004). Conformational flexibility in recombinant measles virus nucleocapsids visualised by cryo-negative stain electron microscopy and real-space helical reconstruction.. J Mol Biol.

[11] Halpin K, Bankamp B, Harcourt BH, Bellini WJ, Rota PA (2004). Nipah virus conforms to the rule of six in a minigenome replication assay.. J Gen Virol.

[12] Park MS, Shaw ML, Munoz-Jordan J, Cros JF, Nakaya T (2003). Newcastle disease virus (NDV)-based assay demonstrates interferon-antagonist activity for the NDV V protein and the Nipah virus V, W, and C proteins.. J Virol.

[13] Sleeman K, Bankamp B, Hummel KB, Lo MK, Bellini WJ (2008). The C, V and W proteins of Nipah virus inhibit minigenome replication.. J Gen Virol.

[14] Lou Z, Xu Y, Xiang K, Su N, Qin L (2006). Crystal structures of Nipah and Hendra virus fusion core proteins.. Febs J.

[15] Bowden TA, Crispin M, Harvey DJ, Aricescu AR, Grimes JM (2008). Crystal structure and carbohydrate analysis of Nipah virus attachment glycoprotein: a template for antiviral and vaccine design.. J Virol.

[16] Bowden TA, Aricescu AR, Gilbert RJ, Grimes JM, Jones EY (2008). Structural basis of Nipah and Hendra virus attachment to their cell-surface receptor ephrin-B2.. Nat Struct Mol Biol.

[17] Bowden TA, Crispin M, Harvey DJ, Jones EY, Stuart DI Dimeric (2010). Architecture of the Hendra Virus Attachment Glycoprotein: Evidence for a Conserved Mode of Assembly.. J Virol.

[18] Karlin D, Longhi S, Receveur V, Canard B (2002). The N-terminal domain of the phosphoprotein of morbilliviruses belongs to the natively unfolded class of proteins.. Virology.

[19] Bourhis J, Johansson K, Receveur-Bréchot V, Oldfield CJ, Dunker AK (2004). The C-terminal domain of measles virus nucleoprotein belongs to the classof intrinsically disordered proteins that fold upon binding to their pohysiological partner.. Virus Research.

[20] Bourhis JM, Receveur-Bréchot V, Oglesbee M, Zhang X, Buccellato M (2005). The intrinsically disordered C-terminal domain of the measles virus nucleoprotein interacts with the C-terminal domain of the phosphoprotein via two distinct sites and remains predominantly unfolded.. Protein Sci.

[21] Bourhis JM, Canard B, Longhi S (2005). Désordre structural au sein du complexe réplicatif du virus de la rougeole: implications fonctionnelles.. Virologie.

[22] Bourhis JM, Canard B, Longhi S (2006). Structural disorder within the replicative complex of measles virus: functional implications.. Virology.

[23] Bourhis JM, Longhi S, Longhi S (2007). Measles virus nucleoprotein: structural organization and functional role of the intrinsically disordered C-terminal domain.. Measles virus nucleoprotein.

[24] Longhi S (2009). Nucleocapsid structure and function.. Curr Top Microbiol Immunol.

[25] Bernard C, Gely S, Bourhis JM, Morelli X, Longhi S (2009). Interaction between the C-terminal domains of N and P proteins of measles virus investigated by NMR.. FEBS Lett.

[26] Karlin D, Ferron F, Canard B, Longhi S (2003). Structural disorder and modular organization in Paramyxovirinae N and P.. J Gen Virol.

[27] Wright PE, Dyson HJ (1999). Intrinsically unstructured proteins: re-assessing the protein structure-function paradigm.. J Mol Biol.

[28] Dunker AK, Oldfield CJ, Meng J, Romero P, Yang JY (2008). The unfoldomics decade: an update on intrinsically disordered proteins.. BMC Genomics.

[29] Dunker AK, Silman I, Uversky VN, Sussman JL (2008). Function and structure of inherently disordered proteins.. Curr Opin Struct Biol.

[30] Dunker AK, Lawson JD, Brown CJ, Williams RM, Romero P (2001). Intrinsically disordered protein.. J Mol Graph Model.

[31] Dunker AK, Obradovic Z (2001). The protein trinity–linking function and disorder.. Nat Biotechnol.

[32] Uversky VN (2002). Natively unfolded proteins: a point where biology waits for physics.. Protein Sci.

[33] Uversky VN (2003). Protein folding revisited. A polypeptide chain at the folding-misfolding-nonfolding cross-roads: which way to go?. Cell Mol Life Sci.

[34] Dyson HJ, Wright PE (2005). Intrinsically unstructured proteins and their functions.. Nat Rev Mol Cell Biol.

[35] Radivojac P, Iakoucheva LM, Oldfield CJ, Obradovic Z, Uversky VN (2007). Intrinsic disorder and functional proteomics.. Biophys J.

[36] Lieutaud P, Canard B, Longhi S (2008). MeDor: a metaserver for predicting protein disorder.. BMC Genomics.

[37] Romero P, Obradovic Z, Li X, Garner EC, Brown CJ (2001). Sequence complexity of disordered proteins.. Proteins.

[38] Brown CJ, Takayama S, Campen AM, Vise P, Marshall TW (2002). Evolutionary rate heterogeneity in proteins with long disordered regions.. J Mol Evol.

[39] Liu J, Tan H, Rost B (2002). Loopy proteins appear conserved in evolution.. J Mol Biol.

[40] Ferron F, Rancurel C, Longhi S, Cambillau C, Henrissat B (2005). VaZyMolO: a tool to define and classify modularity in viral proteins.. J Gen Virol.

[41] Bourhis JM, Canard B, Longhi S (2007). Predicting protein disorder and induced folding: from theoretical principles to practical applications.. Curr Protein Pept Sci.

[42] Johansson K, Bourhis JM, Campanacci V, Cambillau C, Canard B (2003). Crystal structure of the measles virus phosphoprotein domain responsible for the induced folding of the C-terminal domain of the nucleoprotein.. J Biol Chem.

[43] Blanchard L, Tarbouriech N, Blackledge M, Timmins P, Burmeister WP (2004). Structure and dynamics of the nucleocapsid-binding domain of the Sendai virus phosphoprotein in solution.. Virology.

[44] Kingston RL, Gay LS, Baase WS, Matthews BW (2008). Structure of the nucleocapsid-binding domain from the mumps virus polymerase; an example of protein folding induced by crystallization.. J Mol Biol.

[45] Uversky VN, Gillespie JR, Fink AL (2000). Why are “natively unfolded” proteins unstructured under physiologic conditions?. Proteins.

[46] Tompa P (2002). Intrinsically unstructured proteins.. Trends Biochem Sci.

[47] Receveur-Bréchot V, Bourhis JM, Uversky VN, Canard B, Longhi S (2006). Assessing protein disorder and induced folding.. Proteins: Structure, Function and Bioinformatics.

[48] Fontana A, Polverino de Laureto P, De Filippis V, Scaramella E, Zambonin M (1997). Probing the partly folded states of proteins by limited proteolysis.. Fold Des.

[49] Hubbard SJ (1998). The structural aspects of limited proteolysis of native proteins.. Biochim Biophys Acta.

[50] Uversky VN (2002). What does it mean to be natively unfolded?. Eur J Biochem.

[51] Uversky VN (1993). Use of fast protein size-exclusion liquid chromatography to study the unfolding of proteins which denature through the molten globule.. Biochemistry.

[52] Wu J, Yang JT, Wu CS (1992). Beta-II conformation of all-beta proteins can be distinguished from unordered form by circular dichroism.. Anal Biochem.

[53] Tell G, Perrone L, Fabbro D, Pellizzari L, Pucillo C (1998). Structural and functional properties of the N transcriptional activation domain of thyroid transcription factor-1: similarities with the acidic activation domains.. Biochem J.

[54] Hua QX, Jia WH, Bullock BP, Habener JF, Weiss MA (1998). Transcriptional activator-coactivator recognition: nascent folding of a kinase-inducible transactivation domain predicts its structure on coactivator binding.. Biochemistry.

[55] Dahlman-Wright K, McEwan IJ (1996). Structural studies of mutant glucocorticoid receptor transactivation domains establish a link between transactivation activity in vivo and alpha-helix-forming potential in vitro.. Biochemistry.

[56] Brocca S, Samalikova M, Uversky VN, Lotti M, Vanoni M (2009). Order propensity of an intrinsically disordered protein, the cyclin-dependent-kinase inhibitor Sic1.. Proteins.

[57] Soulages JL, Kim K, Walters C, Cushman JC (2002). Temperature-induced extended helix/random coil transitions in a group 1 late embryogenesis-abundant protein from soybean.. Plant Physiol.

[58] Uversky VN, Uversky VN, Longhi S (2010). Analyzing intrinsically disordered proteins by size exclusion chromatography.. Instrumental analysis of intrinsically disordered proteins.

[59] Csizmok V, Szollosi E, Friedrich P, Tompa P (2006). A novel two-dimensional electrophoresis technique for the identification of intrinsically unstructured proteins.. Mol Cell Proteomics.

[60] Oldfield CJ, Cheng Y, Cortese MS, Romero P, Uversky VN (2005). Coupled Folding and Binding with alpha-Helix-Forming Molecular Recognition Elements.. Biochemistry.

[61] Mohan A, Oldfield CJ, Radivojac P, Vacic V, Cortese MS (2006). Analysis of Molecular Recognition Features (MoRFs).. J Mol Biol.

[62] Vacic V, Oldfield CJ, Mohan A, Radivojac P, Cortese MS (2007). Characterization of molecular recognition features, MoRFs, and their binding partners.. J Proteome Res.

[63] Fuxreiter M, Tompa P, Simon I (2007). Local structural disorder imparts plasticity on linear motifs.. Bioinformatics.

[64] Fuxreiter M, Simon I, Friedrich P, Tompa P (2004). Preformed structural elements feature in partner recognition by intrinsically unstructured proteins.. J Mol Biol.

[65] Lacy ER, Filippov I, Lewis WS, Otieno S, Xiao L (2004). p27 binds cyclin-CDK complexes through a sequential mechanism involving binding-induced protein folding.. Nat Struct Mol Biol.

[66] Sivakolundu SG, Bashford D, Kriwacki RW (2005). Disordered p27Kip1 exhibits intrinsic structure resembling the Cdk2/cyclin A-bound conformation.. J Mol Biol.

[67] Van Hoy M, Leuther KK, Kodadek T, Johnston SA (1993). The acidic activation domains of the GCN4 and GAL4 proteins are not alpha helical but form beta sheets.. Cell.

[68] Belle V, Rouger S, Costanzo S, Liquiere E, Strancar J (2008). Mapping alpha-helical induced folding within the intrinsically disordered C-terminal domain of the measles virus nucleoprotein by site-directed spin-labeling EPR spectroscopy.. Proteins: Structure, Function and Bioinformatics.

[69] Callebaut I, Labesse G, Durand P, Poupon A, Canard L (1997). Deciphering protein sequence information through hydrophobic cluster analysis (HCA): current status and perspectives.. Cell Mol Life Sci.

[70] Morin B, Bourhis JM, Belle V, Woudstra M, Carrière F (2006). Assessing induced folding of an intrinsically disordered protein by site-directed spin-labeling EPR spectroscopy.. J Phys Chem B.

[71] Gely S, Lowry DF, Bernard C, Ringkjobing-Jensen M, Blackledge M (2010). Solution structure of the C-terminal X domain of the measles virus phosphoprotein and interaction with the intrinsically disordered C-terminal domain of the nucleoprotein.. J Mol Recognit.

[72] Kavalenka A, Urbancic I, Belle V, Rouger S, Costanzo S (2010). Conformational analysis of the partially disordered measles virus NTAIL-XD complex by SDSL EPR spectroscopy.. Biophys J.

[73] Houben K, Marion D, Tarbouriech N, Ruigrok RW, Blanchard L (2007). Interaction of the C-terminal domains of sendai virus N and P proteins: comparison of polymerase-nucleocapsid interactions within the paramyxovirus family.. J Virol.

[74] Jordan IK, Sutter BA, McClure MA (2000). Molecular evolution of the Paramyxoviridae and Rhabdoviridae multiple-protein-encoding P gene.. Mol Biol Evol.

[75] Narechania A, Terai M, Burk RD (2005). Overlapping reading frames in closely related human papillomaviruses result in modular rates of selection within E2.. J Gen Virol.

[76] Rancurel C, Khosravi M, Dunker KA, Romero PR, Karlin D (2009). Overlapping genes produce proteins with unusual sequence properties and offer insight into de novo protein creation.. J Virol.

[77] Kovacs E, Tompa P, Liliom K, Kalmar L Dual coding in alternative reading frames correlates with intrinsic protein disorder.. Proc Natl Acad Sci U S A.

[78] Boumlic A, Nomine Y, Charbonnier S, Dalagiorgou G, Vassilaki N Prevalence of intrinsic disorder in the hepatitis C virus ARFP/Core+1/S protein.. Febs J.

[79] Dunker AK, Cortese MS, Romero P, Iakoucheva LM, Uversky VN (2005). Flexible nets.. Febs J.

[80] Uversky VN, Oldfield CJ, Dunker AK (2005). Showing your ID: intrinsic disorder as an ID for recognition, regulation and cell signaling.. J Mol Recognit.

[81] Haynes C, Oldfield CJ, Ji F, Klitgord N, Cusick ME (2006). Intrinsic disorder is a common feature of hub proteins from four eukaryotic interactomes.. PLoS Comput Biol.

[82] Dunker AK, Garner E, Guilliot S, Romero P, Albrecht K (1998). Protein disorder and the evolution of molecular recognition: theory, predictions and observations.. Pac Symp Biocomput.

[83] Dunker AK, Brown CJ, Obradovic Z (2002). Identification and functions of usefully disordered proteins.. Adv Protein Chem.

[84] Uversky VN, Li J, Souillac P, Jakes R, Goedert M (2002). Biophysical properties of the synucleins and their propensities to fibrillate: inhibition of alpha-synuclein assembly by beta- and gamma- synucleins.. J Biol Chem.

[85] Gunasekaran K, Tsai CJ, Kumar S, Zanuy D, Nussinov R (2003). Extended disordered proteins: targeting function with less scaffold.. Trends Biochem Sci.

[86] Fink AL (2005). Natively unfolded proteins.. Curr Opin Struct Biol.

[87] Zhang X, Glendening C, Linke H, Parks CL, Brooks C (2002). Identification and characterization of a regulatory domain on the carboxyl terminus of the measles virus nucleocapsid protein.. J Virol.

[88] Zhang X, Bourhis JM, Longhi S, Carsillo T, Buccellato M (2005). Hsp72 recognizes a P binding motif in the measles virus N protein C-terminus.. Virology.

[89] Couturier M, Buccellato M, Costanzo S, Bourhis JM, Shu Y (2009). High Affinity Binding between Hsp70 and the C-Terminal Domain of the Measles Virus Nucleoprotein Requires an Hsp40 Co-Chaperone.. J Mol Recognit.

[90] tenOever BR, Servant MJ, Grandvaux N, Lin R, Hiscott J (2002). Recognition of the Measles Virus Nucleocapsid as a Mechanism of IRF-3 Activation.. J Virol.

[91] Colombo M, Bourhis JM, Chamontin C, Soriano C, Villet S (2009). The interaction between the measles virus nucleoprotein and the Interferon Regulator Factor 3 relies on a specific cellular environment.. Virol J.

[92] Laine D, Bourhis J, Longhi S, Flacher M, Cassard L (2005). Measles virus nucleoprotein induces cell proliferation arrest and apoptosis through NTAIL/NR and NCORE/FcgRIIB1 interactions, respectively.. J Gen Virol.

[93] Laine D, Trescol-Biémont M, Longhi S, Libeau G, Marie J (2003). Measles virus nucleoprotein binds to a novel cell surface receptor distinct from FcgRII via its C-terminal domain: role in MV-induced immunosuppression.. J Virol.

[94] Sato H, Masuda M, Miura R, Yoneda M, Kai C (2006). Morbillivirus nucleoprotein possesses a novel nuclear localization signal and a CRM1-independent nuclear export signal.. Virology.

[95] Iwasaki M, Takeda M, Shirogane Y, Nakatsu Y, Nakamura T (2009). The matrix protein of measles virus regulates viral RNA synthesis and assembly by interacting with the nucleocapsid protein.. J Virol.

[96] De BP, Banerjee AK (1999). Involvement of actin microfilaments in the transcription/replication of human parainfluenza virus type 3: possible role of actin in other viruses.. Microsc Res Tech.

[97] Moyer SA, Baker SC, Horikami SM (1990). Host cell proteins required for measles virus reproduction.. J Gen Virol.

[98] Chen M, Cortay JC, Gerlier D (2003). Measles virus protein interactions in yeast: new findings and caveats.. Virus Res.

[99] Liston P, DiFlumeri C, Briedis DJ (1995). Protein interactions entered into by the measles virus P, V, and C proteins.. Virus Res.

[100] Curran J, Pelet T, Kolakofsky D (1994). An acidic activation-like domain of the Sendai virus P protein is required for RNA synthesis and encapsidation.. Virology.

[101] Curran J, Marq JB, Kolakofsky D (1995). An N-terminal domain of the Sendai paramyxovirus P protein acts as a chaperone for the NP protein during the nascent chain assembly step of genome replication.. J Virol.

[102] Watanabe N, Kawano M, Tsurudome M, Nishio M, Ito M (1996). Binding of the V proteins to the nucleocapsid proteins of human parainfluenza type 2 virus.. Med Microbiol Immunol (Berl).

[103] Chinchar VG, Portner A (1981). Functions of Sendai virus nucleocapsid polypeptides: enzymatic activities in nucleocapsids following cleavage of polypeptide P by Staphylococcus aureus protease V8.. Virology.

[104] Deshpande KL, Portner A (1985). Monoclonal antibodies to the P protein of Sendai virus define its structure and role in transcription.. Virology.

[105] Tarbouriech N, Curran J, Ebel C, Ruigrok RW, Burmeister WP (2000). On the domain structure and the polymerization state of the sendai virus P protein.. Virology.

[106] Bernado P, Blanchard L, Timmins P, Marion D, Ruigrok RW (2005). A structural model for unfolded proteins from residual dipolar couplings and small-angle x-ray scattering.. Proc Natl Acad Sci U S A.

[107] Houben K, Blanchard L, Blackledge M, Marion D (2007). Intrinsic dynamics of the partly unstructured PX domain from the Sendai virus RNA polymerase cofactor P.. Biophys J.

[108] Dedmon MM, Patel CN, Young GB, Pielak GJ (2002). FlgM gains structure in living cells.. Proc Natl Acad Sci U S A.

[109] McNulty BC, Young GB, Pielak GJ (2006). Macromolecular crowding in the Escherichia coli periplasm maintains alpha-synuclein disorder.. J Mol Biol.

[110] Bodart JF, Wieruszeski JM, Amniai L, Leroy A, Landrieu I (2008). NMR observation of Tau in Xenopus oocytes.. J Magn Reson.

[111] Tokuriki N, Oldfield CJ, Uversky VN, Berezovsky IN, Tawfik DS (2009). Do viral proteins possess unique biophysical features?. Trends Biochem Sci.

[112] Cheng Y, Legall T, Oldfield CJ, Mueller JP, Van YY (2006). Rational drug design via intrinsically disordered protein.. Trends Biotechnol.

[113] Larkin MA, Blackshields G, Brown NP, Chenna R, McGettigan PA (2007). Clustal W and Clustal X version 2.0.. Bioinformatics.

[114] Gouet P, Courcelle E, Stuart DI, Metoz F (1999). ESPript: analysis of multiple sequence alignments in PostScript.. Bioinformatics.

[115] Bryson K, McGuffin LJ, Marsden RL, Ward JJ, Sodhi JS (2005). Protein structure prediction servers at University College London.. Nucleic Acids Res.

[116] Dosztanyi Z, Csizmok V, Tompa P, Simon I (2005). IUPred: web server for the prediction of intrinsically unstructured regions of proteins based on estimated energy content.. Bioinformatics.

[117] Coeytaux K, Poupon A (2005). Prediction of unfolded segments in a protein sequence based on amino acid composition.. Bioinformatics.

[118] Yang ZR, Thomson R, McNeil P, Esnouf RM (2005). RONN: the bio-basis function neural network technique applied to the detection of natively disordered regions in proteins.. Bioinformatics.

[119] Galzitskaya OV, Garbuzynskiy SO, Lobanov MY (2006). FoldUnfold: web server for the prediction of disordered regions in protein chain.. Bioinformatics.

[120] Garbuzynskiy SO, Lobanov MY, Galzitskaya OV (2004). To be folded or to be unfolded?. Protein Sci.

[121] Linding R, Jensen LJ, Diella F, Bork P, Gibson TJ (2003). Protein disorder prediction: implications for structural proteomics.. Structure (Camb).

[122] Prilusky J, Felder CE, Zeev-Ben-Mordehai T, Rydberg EH, Man O (2005). FoldIndex: a simple tool to predict whether a given protein sequence is intrinsically unfolded.. Bioinformatics.

[123] Linding R, Russell RB, Neduva V, Gibson TJ (2003). GlobPlot: Exploring protein sequences for globularity and disorder.. Nucleic Acids Res.

[124] Obradovic Z, Peng K, Vucetic S, Radivojac P, Brown CJ (2003). Predicting intrinsic disorder from amino acid sequence.. Proteins.

[125] Obradovic Z, Peng K, Vucetic S, Radivojac P, Dunker AK (2005). Exploiting heterogeneous sequence properties improves prediction of protein disorder.. Proteins.

[126] Chandonia JM, Karplus M (1999). New methods for accurate prediction of protein secondary structure.. Proteins.

[127] Ferron F, Longhi S, Canard B, Karlin D (2006). A practical overview of protein disorder prediction methods.. Proteins.

[128] Dosztanyi Z, Sandor M, Tompa P, Simon I (2007). Prediction of protein disorder at the domain level.. Curr Protein Pept Sci.

[129] He B, Wang K, Liu Y, Xue B, Uversky VN (2009). Predicting intrinsic disorder in proteins: an overview.. Cell Res.

[130] Lupas A, Van Dyke M, Stock J (1991). Predicting coiled coils from protein sequences.. Science.

[131] Kelley LA, MacCallum RM, Sternberg MJ (2000). Enhanced genome annotation using structural profiles in the program 3D-PSSM.. J Mol Biol.

[132] Vacic V, Uversky VN, Dunker AK, Lonardi S (2007). Composition Profiler: a tool for discovery and visualization of amino acid composition differences.. BMC Bioinformatics.

[133] Wootton JC (1994). Non-globular domains in protein sequences: automated segmentation using complexity measures.. Comput Chem.

[134] Hwang TL, Shaka AJ (1998). Multiple-pulse mixing sequences that selectively enhance chemical exchange or cross-relaxation peaks in high-resolution NMR spectra.. J Magn Reson.

[135] Whitmore L, Wallace BA (2004). DICHROWEB, an online server for protein secondary structure analyses from circular dichroism spectroscopic data.. Nucleic Acids Res.

[136] Whitmore L, Wallace BA (2008). Protein secondary structure analyses from circular dichroism spectroscopy: methods and reference databases.. Biopolymers.

[137] Kawahara K, Tanford C (1966). Viscosity and density of aqueous solutions of urea and guanidine hydrochloride.. J Biol Chem.

[138] Campen A, Williams RM, Brown CJ, Meng J, Uversky VN (2008). TOP-IDP-scale: a new amino acid scale measuring propensity for intrinsic disorder.. Protein Pept Lett.

