# Supplementary figures and images for: Structural Disorder within Henipavirus Nucleoprotein and Phosphoprotein: From Predictions to Experimental Assessment
Source: PLoS One. 2010 Jul 21;5(7):e11684. doi: 10.1371/journal.pone.0011684 (PMC2908138; doi:10.1371/journal.pone.0011684)

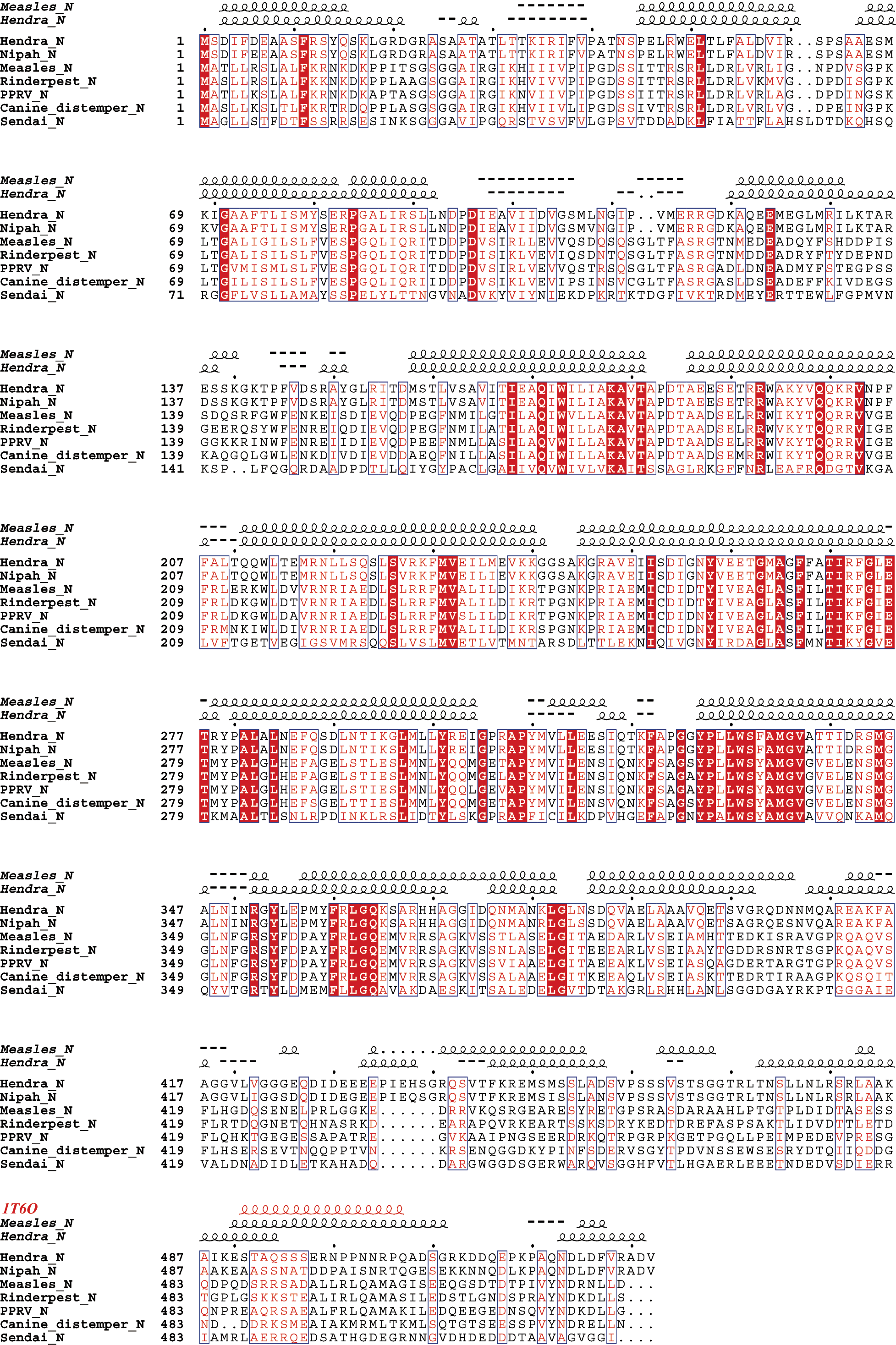

Supplement: Figure S1 — Multiple sequence alignment of Henipavirus, Morbillivirus and Respirovirus N proteins as obtained using ClustalW [113] (http://www.ebi.ac.uk/Tools/clustalw2/index.html) and ESPript [114] (http://espript.ibcp.fr/ESPript/cgi-bin/ESPript.cgi). Residues corresponding to a similarity above 60% are boxed and shown in red. Identical residues are boxed and shown in white on a red background. The front numbers correspond to the amino acid position in sequence. Dots above the alignment indicate intervals of 10 residues. Predicted secondary structure elements, as obtained using the PSIPRED server [115] (http://bioinf.cs.ucl.ac.uk/psipred/), for Hendra and Measles virus N are shown above the multiple sequence alignment. The red helix spanning residues 487–503 of Measles virus N corresponds to the helical segment observed in the crystal structure of a chimeric construct consisting of the C-terminal domain of the Measles virus P protein and of residues 486–504 of N (pdb code : 1T6O). The accession numbers of the N proteins are: NP 047106.1 (Hendra virus), NP 112021.1 (Nipah virus), Q89933.1 (measles virus), ABM64790.1 (Rinderpest virus), ABY61984.1 (Peste des Petits Ruminants virus), BAI60055.1 (Canine Distemper virus), AAB06278.1 (Sendai virus). A significant sequence divergence can be observed starting from position 400 up to the C-terminus. (0.99 MB DOC) [file pone.0011684.s001.doc]

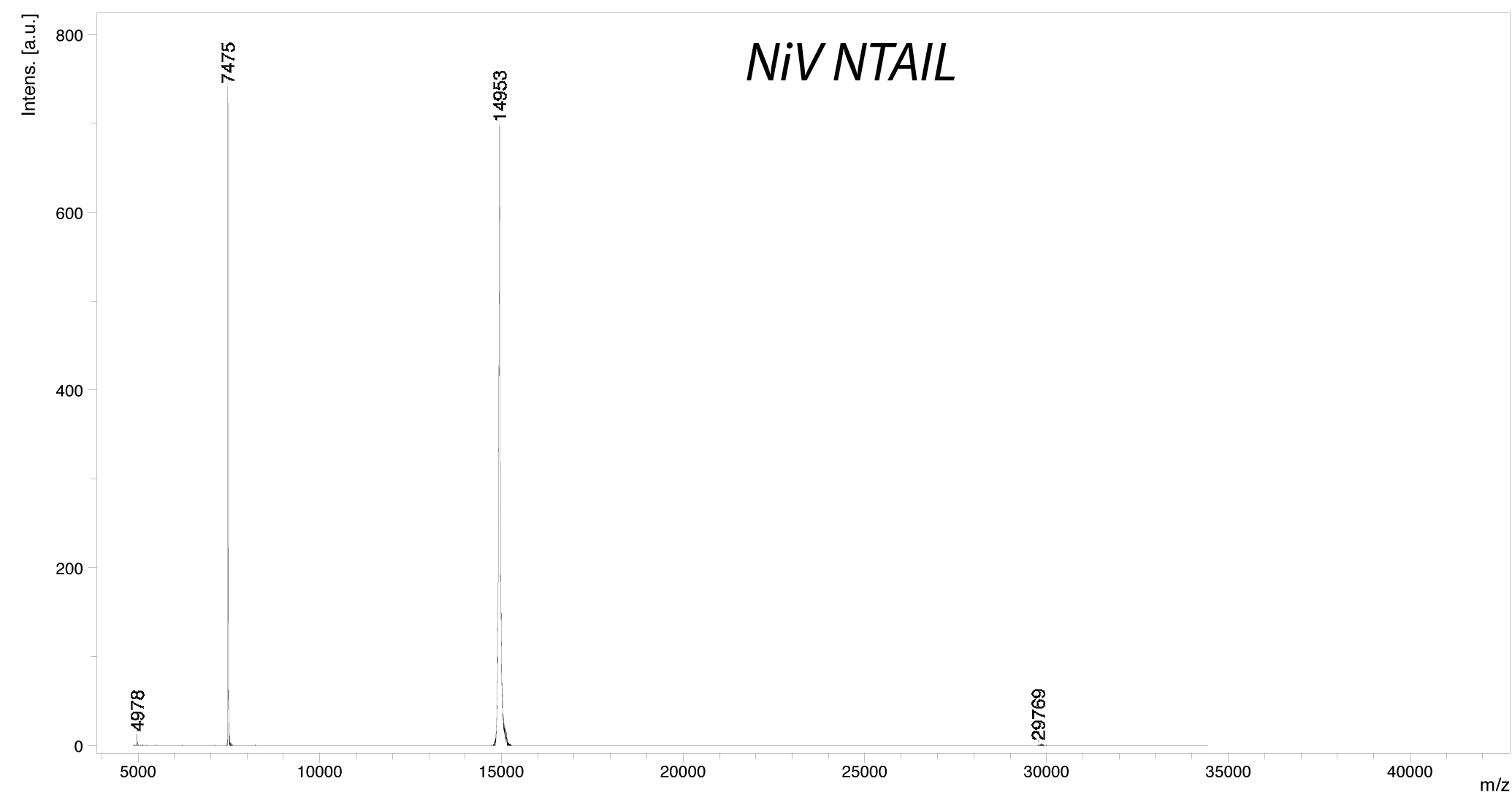

Supplement: Figure S2 — Mass spectrometry (MALDI-TOF) analysis of recombinant, hexahistidine tagged NiV NTAIL purified from the soluble fraction of E. coli. Mass analysis was performed using an Autoflex II TOF/TOF. Spectra were acquired in the linear mode. The sample (0.7 µL containing 15 pmol) was mixed with an equal volume of sinapinic acid matrix solution, spotted on the target, then dried at room temperature for 10 min. The mass standard was myoglobin. Proteins were analyzed in the Autoflex matrix-assisted laser desorption ionization/time of flight (Bruker Daltonics, Bremen, Germany). A major peak with a mass slightly higher (14 953 Da) than expected (14 949 Da) was observed. The additional peak of 7 475 Da in mass, very probably corresponds to a degradation product, as the protein was shown to contain no contaminating proteins (as shown by mass spectrometry analysis of trypic fragments). (0.05 MB DOC) [file pone.0011684.s002.doc]

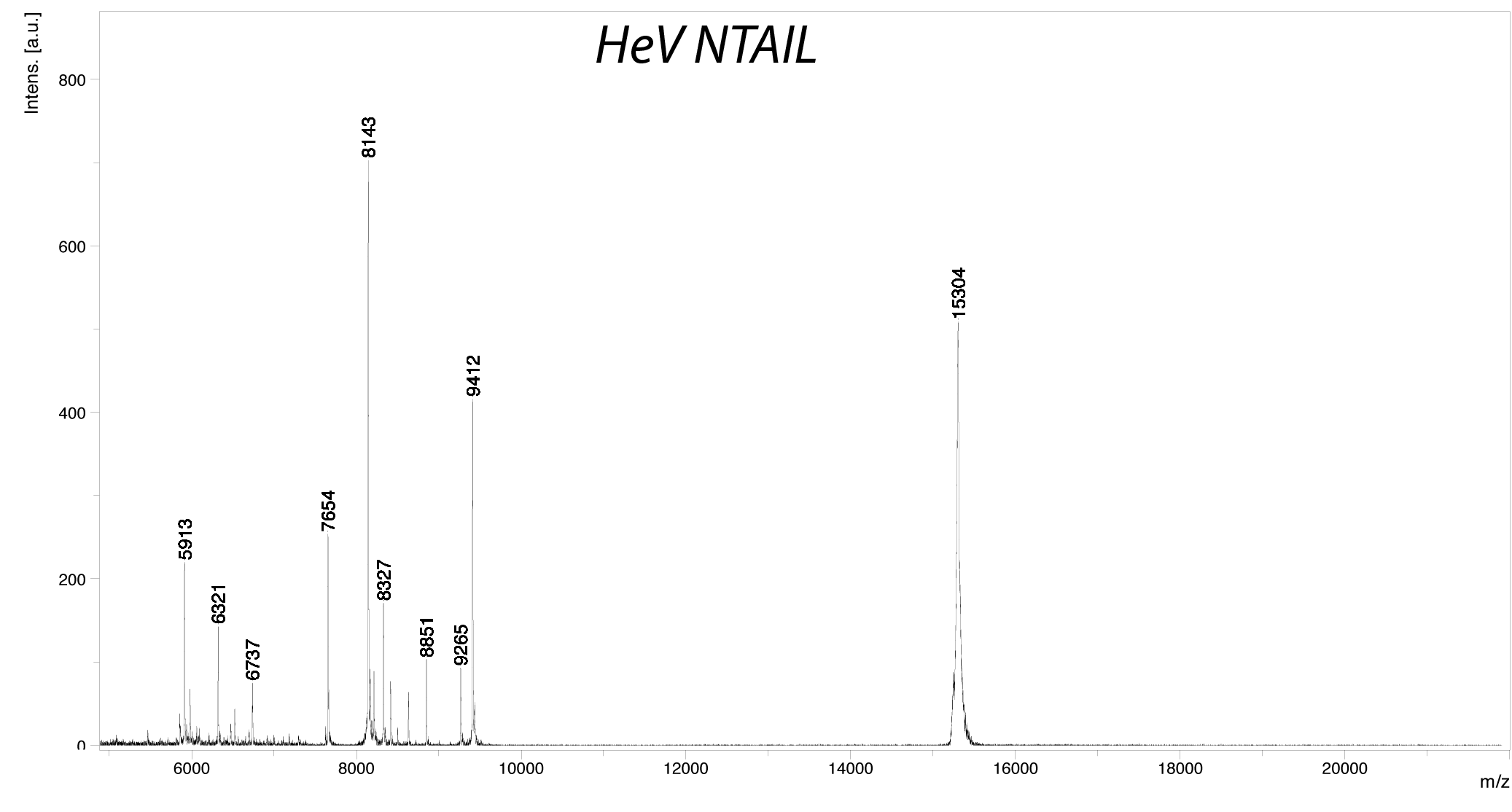

Supplement: Figure S3 — Mass spectrometry (MALDI-TOF) analysis of recombinant, hexahistidine tagged HeV NTAIL purified from the soluble fraction of E. coli. Mass analysis was performed using an Autoflex II TOF/TOF. Spectra were acquired in the linear mode. The sample (0.7 µL containing 15 pmol) was mixed with an equal volume of sinapinic acid matrix solution, spotted on the target, then dried at room temperature for 10 min. The mass standard was myoglobin. Proteins were analyzed in the Autoflex matrix-assisted laser desorption ionization/time of flight (Bruker Daltonics, Bremen, Germany). A peak with a mass slightly higher (15 304 Da) than expected (15 241 Da) was observed. The numerous additional peaks corresponding to species of lower molecular mass likely correspond to degradation products, as the protein was found to be devoid of contaminating protein by mass spectrometry analysis of tryptic fragments. (0.06 MB DOC) [file pone.0011684.s003.doc]

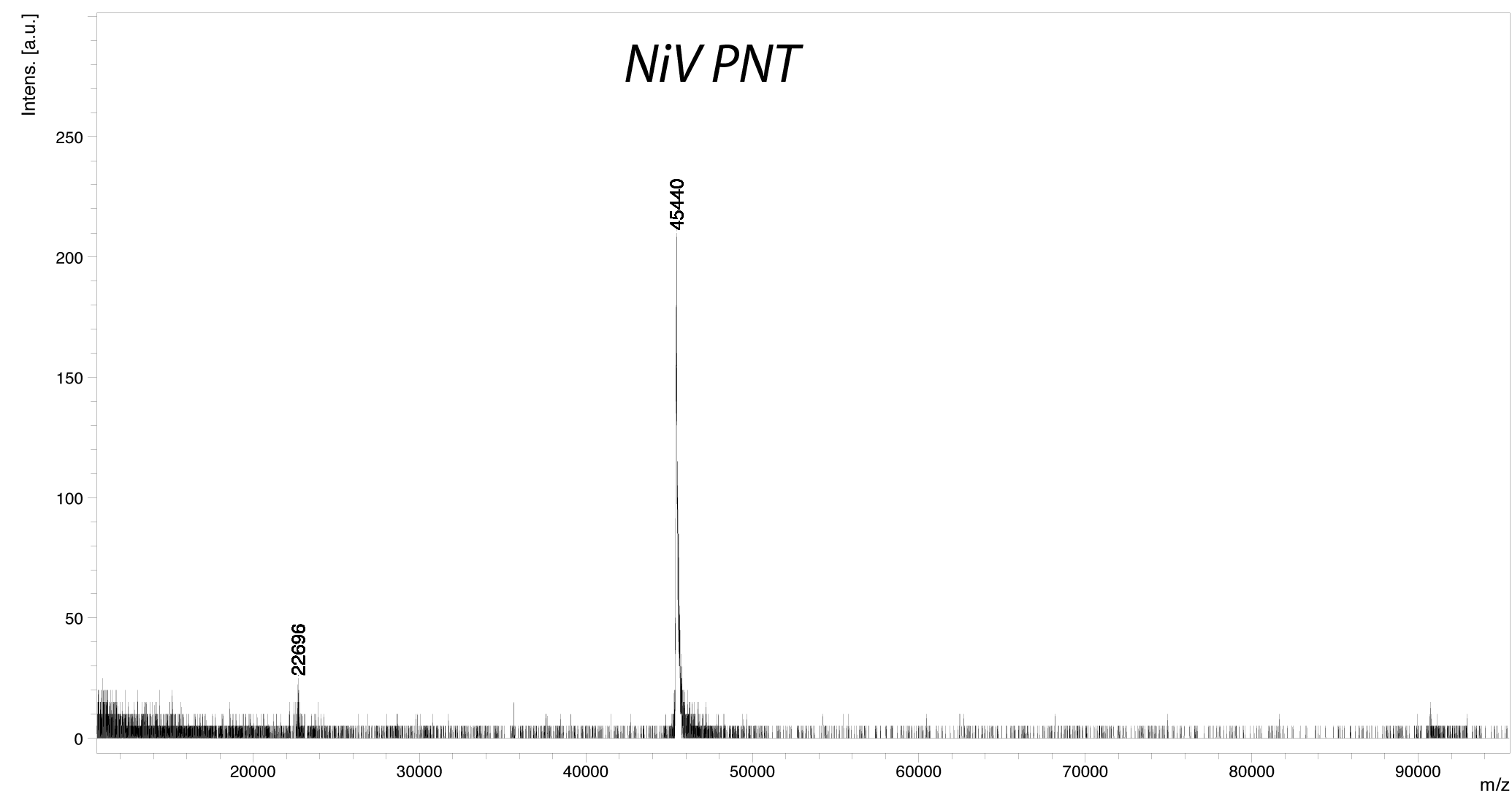

Supplement: Figure S4 — Mass spectrometry (MALDI-TOF) analysis of recombinant, hexahistidine tagged NiV PNT purified from the soluble fraction of E. coli. Mass analysis was performed using an Autoflex II TOF/TOF. Spectra were acquired in the linear mode. The sample (0.7 µL containing 15 pmol) was mixed with an equal volume of sinapinic acid matrix solution, spotted on the target, then dried at room temperature for 10 min. The mass standard was BSA. Proteins were analyzed in the Autoflex matrix-assisted laser desorption ionization/time of flight (Bruker Daltonics, Bremen, Germany). A major peak with a mass slightly higher (45 440 Da) than expected (45 330 Da) was observed. The additional peak (22 696 Da) very probably corresponds to a degradation product, as the protein was found to contain no contaminating proteins (as judged based by mass spectrometry analysis of tryptic fragments). (0.06 MB DOC) [file pone.0011684.s004.doc]

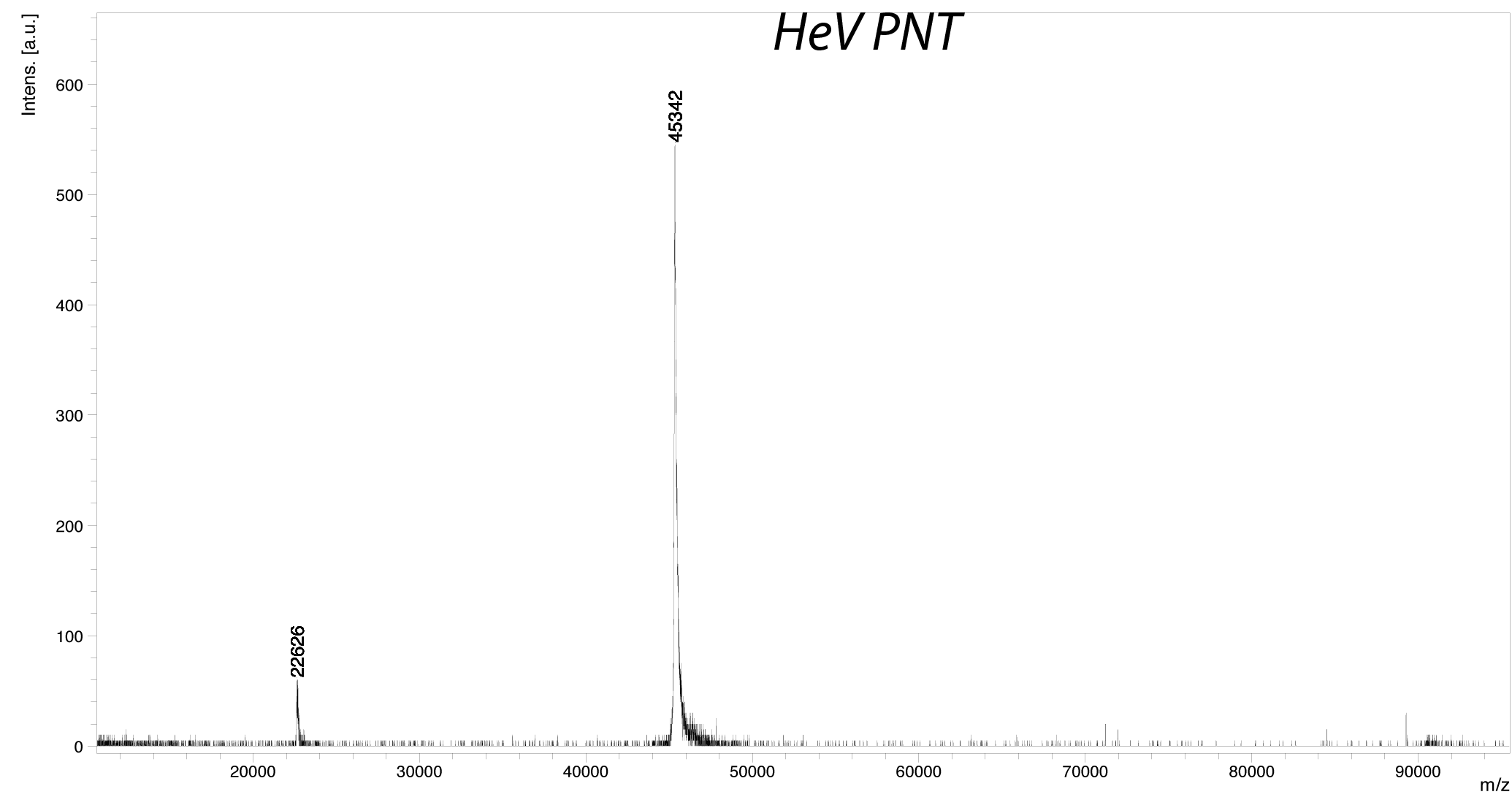

Supplement: Figure S5 — Mass spectrometry (MALDI-TOF) analysis of recombinant, hexahistidine tagged HeV PNT purified from the soluble fraction of E. coli. Mass analysis was performed using an Autoflex II TOF/TOF. Spectra were acquired in the linear mode. The sample (0.7 µL containing 15 pmol) was mixed with an equal volume of sinapinic acid matrix solution, spotted on the target, then dried at room temperature for 10 min. The mass standard was BSA. Proteins were analyzed in the Autoflex matrix-assisted laser desorption ionization/time of flight (Bruker Daltonics, Bremen, Germany). A major peak with a mass is slightly higher (45 342 Da) than expected (45 216 Da) was observed. The additional peak (22626 Da) very probably corresponds to a degradation product, as the protein was found to contain no contaminating proteins (as judged based by mass spectrometry analysis of tryptic fragments). (0.06 MB DOC) [file pone.0011684.s005.doc]

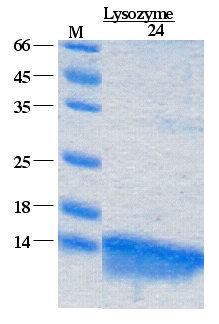

Supplement: Figure S6 — 15% SDS-PAGE analysis of lysozyme after a 24 hours digestion by thermolysin. The digestion was performed by incubating lysozyme (1 mg/mL) with thermolysin in 20 mM Tris/HCl pH 8 at 26°C. The protease∶protein substrate ratio was 1∶100 (w/w). M: molecular markers. No degradation was observed even after an incubation period as long as 24 hours, consistent with the foldedness of the protein. (0.07 MB DOC) [file pone.0011684.s006.doc]
